# De novo design of a novel AIE fluorescent probe tailored to autophagy visualization via pH manipulation

**DOI:** 10.1186/s40824-023-00359-w

**Published:** 2023-03-13

**Authors:** Xueyan Huang, Fei Chen, Yeshuo Ma, Fan Zheng, Yanpeng Fang, Bin Feng, Shuai Huang, Hongliang Zeng, Wenbin Zeng

**Affiliations:** 1 Xiangya School of Pharmaceutical Sciences, Central South University, Changsha, 410013 People’s Republic of China; 2 Hunan Key Laboratory of Diagnostic and Therapeutic Drug Research for Chronic Diseases, Changsha, China; 3 Department of Geriatrics, Third Xiangya Hospital, Central South University, Changsha, People’s Republic of China; 4 Hunan Academic of Chinese Medicine, Inst Chinese Mat Med, Changsha, People’s Republic of China

**Keywords:** Autophagy visualization, Lysosome-targeting, AIE fluorescent probe, Biomaterials imaging

## Abstract

**Background:**

Macroautophagy is an essential cellular self-protection mechanism, and defective autophagy has been considered to contribute to a variety of diseases. During the process, cytoplasmic components are transported via autophagosomes to acidic lysosomes for metabolism and recycling, which represents application niches for lysosome-targeted fluorescent probes. Additionally, in view of the complexity of the autophagy pathway, it entails more stringent requirements for probes suitable for monitoring autophagy. Meanwhile, aggregation-induced emission (AIE) fluorescent probes have been impressively demonstrated in the biomedical field, which bring fascinating possibilities to the autophagy visualization.

**Methods:**

We reported a generalizable de novo design of a novel pH-sensitive AIE probe ASMP-AP tailored to lysosome targeting for the interpretation of autophagy. Firstly, the theoretical calculation was carried out followed by the investigation of optical properties. Then, the performance of ASMP-AP in visualizing autophagy was corroborated by starvation or drugs treatments. Furthermore, the capability of ASMP-AP to monitor autophagy was demonstrated in ex vivo liver tissue and zebrafish in vivo.

**Results:**

ASMP-AP displays a large stokes shift, great cell permeability and good biocompatibility. More importantly, ASMP-AP enables a good linear response to pH, which derives from the fact that its aggregation state can be manipulated by the acidity. It was successfully applied for imaging autophagy in living cells and was proved capable of monitoring mitophagy. Moreover, this novel molecular tool was validated by ex vivo visualization of activated autophagy in drug-induced liver injury model. Interestingly, it provided a meaningful pharmacological insight that the melanin inhibitor 1-phenyl-2-thiourea (PTU)-induced autophagy was clearly presented in wild-type zebrafish.

**Conclusions:**

ASMP-AP offers a simple yet effective tool for studying lysosome and autophagy. This is the first instance to visualize autophagy in zebrafish using a small-molecule probe with AIE characters, accurate lysosome targeting and simultaneous pH sensitivity. Ultimately, this novel fluorescent system has great potential for in vivo translation to fuel autophagy research.

**Graphical Abstract:**

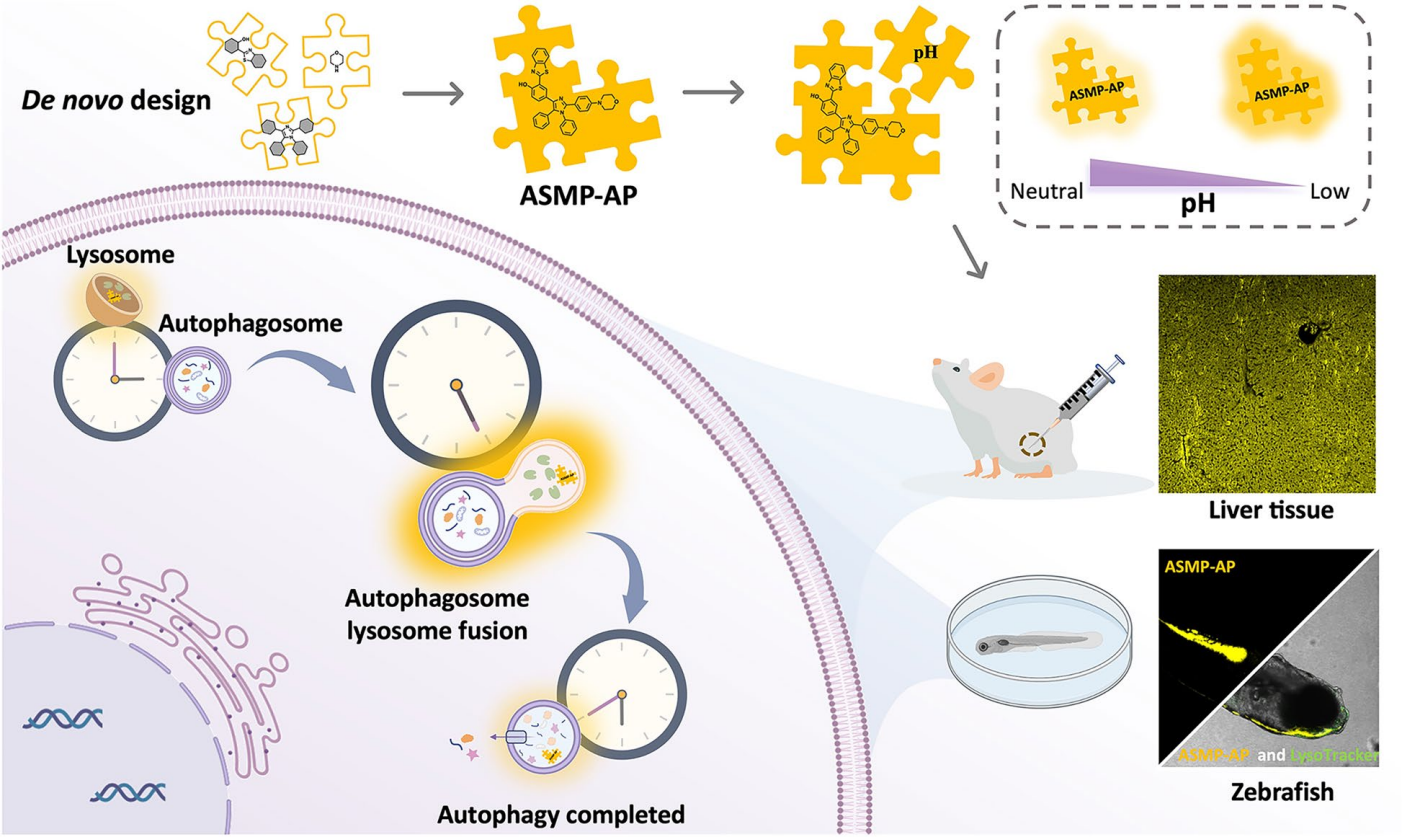

**Supplementary Information:**

The online version contains supplementary material available at 10.1186/s40824-023-00359-w.

## Introduction

Macroautophagy is an evolutionarily conserved and tightly regulated protein degradation process, where the cytoplasmic materials are phagocytized in the newly formed double-membraned autophagosomes and then trafficked to lysosomes for subsequent metabolism and as part of cellular contents renewal [1]. It is usually associated with starvation or other non-physiological stresses, such as molecular material treatments. In these cases, autophagy functions as cellular self-protection. Thus, the defects in autophagy are thought to be implicated in multiple diseases, including cancers [2], protein aggregates related neurodegenerative diseases [3], and Crohn's disease [4]. The visualization of autophagy appears particularly vital to dissect sophisticated autophagy processes and functions and develop new therapeutic strategies. The autophagic pathway relies on lysosomal degradative actions, making the monitoring of lysosome dynamic alterations an attractive target for disease research [5–7]. Indeed, lysosomes have been reported to display definite changes during autophagy, such as increased numbers, larger size, and enhanced acidification [8]. Accordingly, tracking the pH variations of the lysosome is an important part of the visualization of autophagy. To these purposes, fluorescent probes bring fascinating possibilities with their intrinsically distinct advantages of non-invasive imaging and fast information acquisition [9–11].

Up to date, a variety of pH-sensitive fluorescent probes already assist in reporting the alterations of the lysosome [12–17]. They normally connect to weakly basic subunits and trigger changes in fluorescent signal through protonation in the acidic lysosomes. Nevertheless, some limitations still need to be addressed: (i) Protonated fluorescent probes may produce an "alkalizing effect" on lysosomes [18]; (ii) Background interference caused by small Stokes shifts, such as the commonly adopted dyes, rhodamine and cyanine [19]; (iii) Relatively short cellular retention time, resulting in leakage [20]; (iv) Unavailable to detect extremely low pH value. With this in mind, and in view of the complexity of the autophagy pathway, it entails more stringent requirements for probes suitable for monitoring autophagy, which narrows the potential of the reported probes for dynamic characterization. Specifically, fluorescent probes with large Stokes shifts are necessary to avoid overlap between excitation and emission spectra and the resulting self-quenching. The tendency of the probe to dissipate in the stressed cells must also be surmounted. On the other hand, concurrently, some pH-sensitive probes based on different fluorophore skeletons and detection mechanisms that allow imaging of autophagy have been reported. For example, He’s team reported that the pH-dependent iridium complex could be used to track lysosomes during autophagy via the influence of protonation and deprotonation on electronic properties [21]. Wang et al. designed a ICT probe based on coumarin fluorophore for the ratiometric measurement of pH, which was successfully realized imaging drugs-induced cellular autophagy [22]. Wang's group constructed a pH-sensitive probe based on rhodamine skeleton by introducing a novel lysosome targeted unit methylcarbitol, and proved its applicability for monitoring autophagy in vitro [23]. This demonstrated that pH-sensitive probes are of considerable interest in the field of autophagy. Nevertheless, there is an inherent defect of them that inevitably encounter fluorescence quenching in the aqueous media since the aggregation-caused quenching (ACQ) issue, which would be insufficient to support the long-term characterization of the dynamic process autophagy. Aggregation-induced emission (AIE) fluorescent probes have been impressively demonstrated in the biomedical field with the merits of good photobleaching tolerance, high sensitivity, and good biocompatibility [24–29]. The significant fluorescence generated by the aggregates enables long-term observation and little loss of fluorescence signal, thereby guaranteeing a superior alternative to ACQ probes. Briefly, the good photostability and favorable intracellular retention capability provided by AIE behavior could serve to monitor autophagy with higher accuracy. To date, however, only a handful of pH-sensitive AIE probes (Table S1) have been adopted to monitor autophagy [30].

**Scheme 1:**
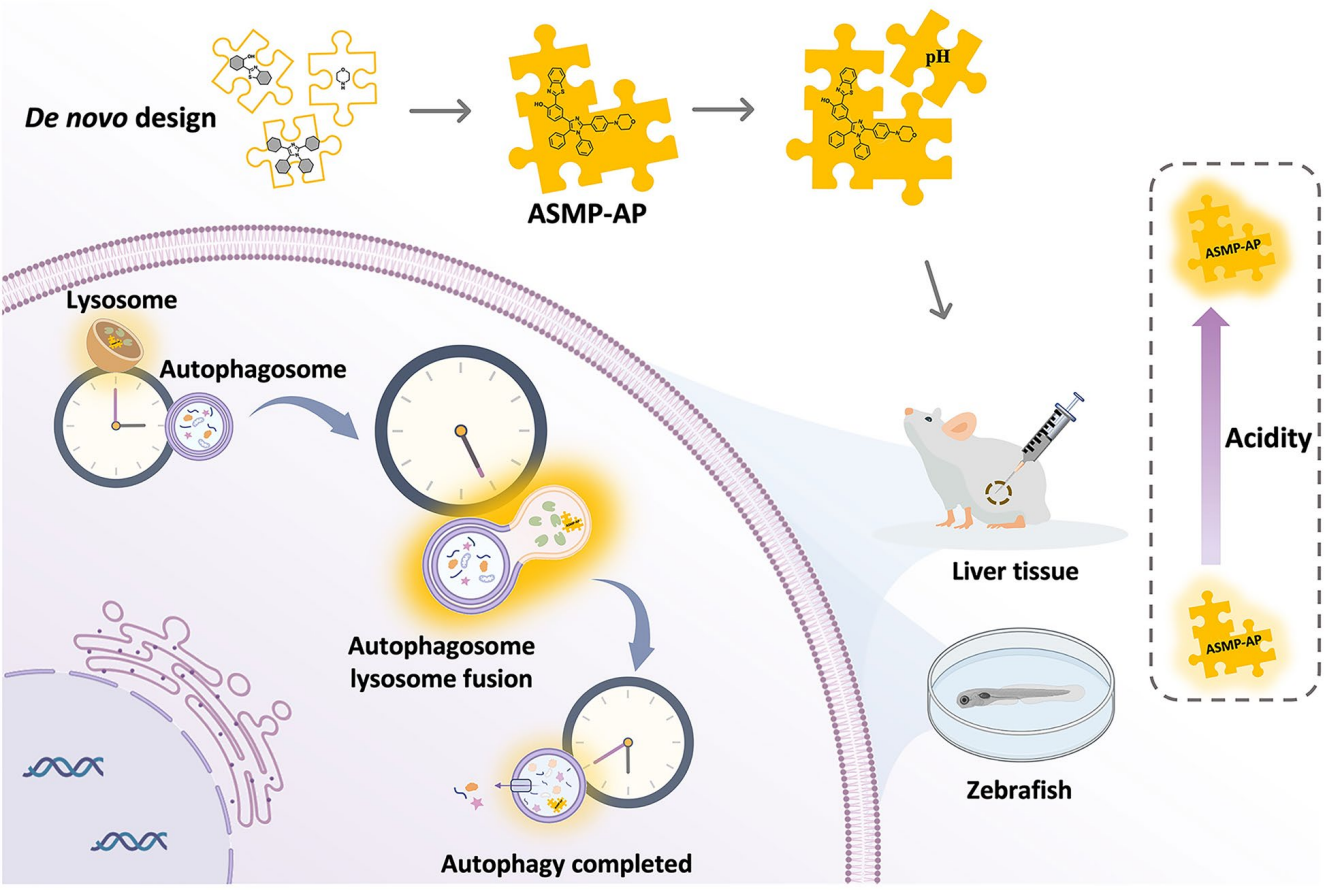
Schematic illustration of autophagy visualization using pH-sensitive AIE fluorescent probe ASMP-AP

Previously, we reported a new approach for the synthesis of asymmetric tetraarylimidazole derivatives, which are well-recognized AIE fluorophores, to facilitate their versatility [31]. In this contribution, by integrating multi-functionality into a single molecule using this synthetic protocol, we de novo designed a novel lysosome targeted AIE probe, 2-(benzo[d]thiazol-2-yl)-4-(2-(4-morpholinophenyl)-1,5-diphenyl-1H-imidazol-4-yl)phenol (**ASMP-AP**), to monitor autophagy that thoughtfully fits the aforementioned demands (Scheme 1). It shows specific lysosomal labeling capability and pH-sensitive optical behavior. In particular, we envisioned that this behavior stems from that acidic pH could finely manipulate the degree of molecular aggregation, which is a rare situation. Notably, it endows the capability to detect the low pH value. The good cell permeability allows for rapid cellular uptake, and subsequently, **ASMP-AP** will be compartmentalized into lysosomes followed by a more compact aggregation state under acidic conditions, thereby generating the significant fluorescence enhancement. Moreover, the autofluorescence overlap of the biomatrix can be reduced due to the relatively large Stokes shift. And owing to the inherent AIE property, **ASMP-AP** can provide a long-term stable and reliable fluorescent signal for autophagy monitoring, which has been well demonstrated in starvation and pharmacological compound-induced autophagy in living cells. Meanwhile, the feasibility to visualize mitophagy has also been proven. We furthermore demonstrated the capability of **ASMP-AP** to detect autophagy at tissue levels. Impressively, the in vivo imaging of zebrafish indicated that treatment of zebrafish with the drug PTU that typically used to inhibit the melanin formation in zebrafish embryos to increase transparency, could induce autophagy to a certain extent. To the best of our knowledge, this is the first paradigm of a small-molecule fluorescent probe in a zebrafish model to elucidate the lysosomal acidification in PTU-induced autophagy. Together, **ASMP-AP** provides a suitable molecular tool for understanding the autophagy process.

## Results

### Design and synthesis

To obtain a novel pH-dependent AIE probe capable of monitoring autophagy, **ASMP-AP** was synthesized via our reported synthetic methods (Scheme S1). There are three key points: (i) An asymmetric tetraarylimidazole acts as luminogen for aggregation-induced emission, which originates from the synergy of the restriction of intramolecular motion (RIM) and ESIPT processes; (ii) The introduction of the classical ESIPT fluorophore 2- (2-hydroxyphenyl)benzothiazole (HBT) guarantees a large Stokes shift; (iii) The morpholine group is elaborately deployed at the beginning of synthesis to confer lysosomal specificity, which exhibits extremely inclusiveness throughout the synthetic routes. This de novo strategy endowed the simply engineerable subcellular localization specificity, which is different from the previous reports [32, 33]. The latter often needs to implement the introduction of functional groups at the last step to achieve the expected fluorescence triggering based on photoinduced electron transfer (PET) or internal charge transfer (ICT) mechanism [14, 34]. The final optical behaviors may deviate from the expectations, which reduces fault tolerance of the probe. Whereas the de novo design can provide greater freedom and expand the chemical space to build a multifunctional fluorescent probes platform. Following the effectively entering cells, the molecules tend to form larger aggregates with the increase of lysosomal acidity, leading to fluorescence intensity increases. This pH-dependent AIE behavior suggests it is a potential probe for tracking lysosomal dynamics. The structure of the **ASMP-AP** was characterized by ^1^H-NMR, ^13^C-NMR and HR-MS (Fig. S16-29, Supporting Information).

### Optical response of ASMP-AP to pH

**Fig. 1:**
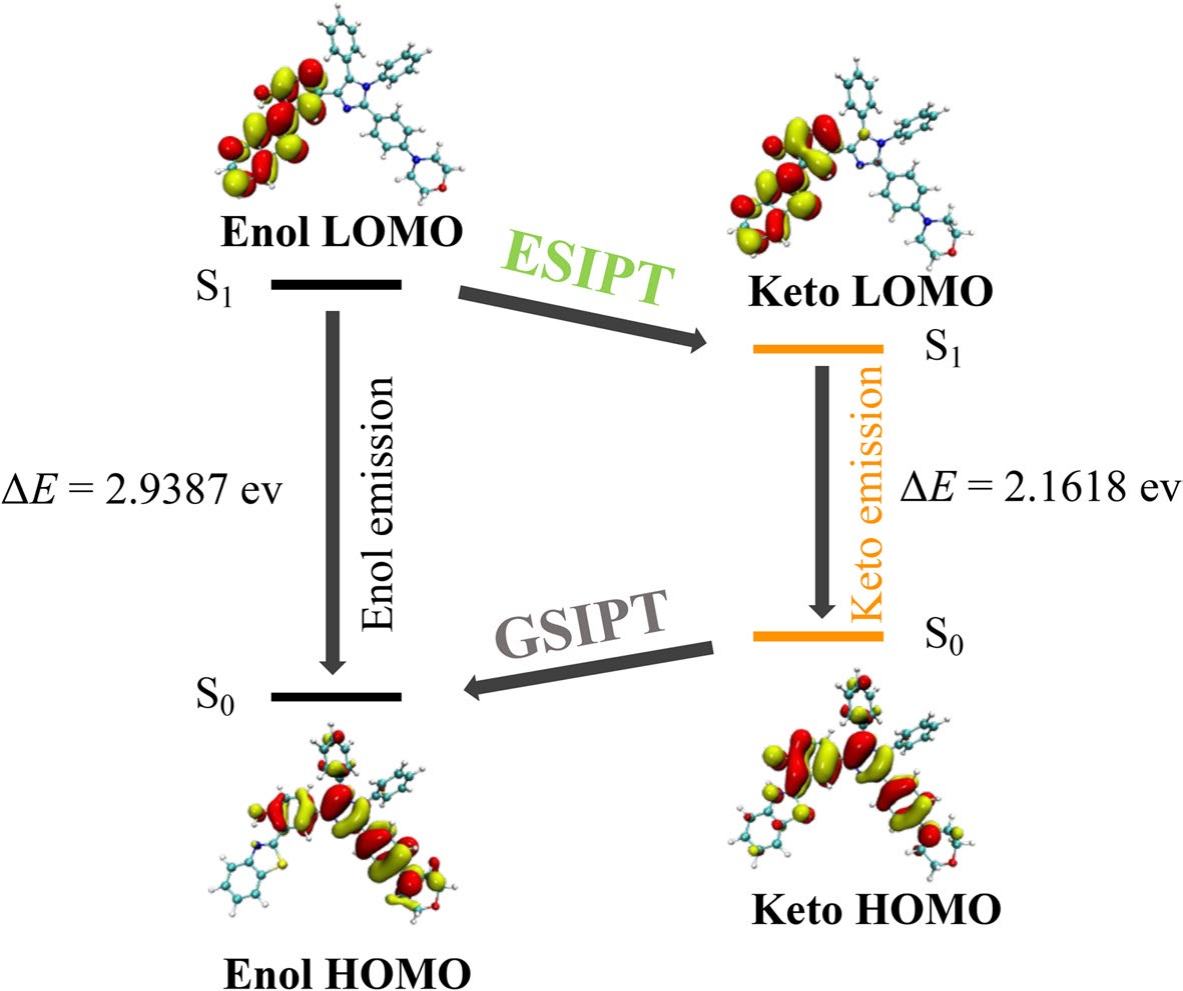
Calculated frontier molecular orbitals of **ASMP-AP** (with LUMO and HOMO) in a four-level diagram

To aid in the grasp on the optical properties of **ASMP-AP**, we carried out theoretical calculation prior to experiments. Firstly, the geometry optimization of probe was conducted via DFT/TDDTF with IEFPCM salvation And a Potential Energy Surface (PES) scan was performed as function of O_7_-H_49_ bond to investigate the ESIPT process. As shown in Fig. S1, ESIPT process was only slightly exergonic for **ASMP-AP** in single molecule state, which means the ESIPT proceeded relatively slow. This is consistent with the study on solvent effect, in which **ASMP-AP** mainly manifested enol state emission (Fig. S2). Then, the information of excited states geometries was provided by frontier molecular orbitals (FMOs) calculation. For intuitive display of the orbitals change, it would be presented with a four-level diagram. As illustrated in Fig. 1, the highest occupied molecular orbital (HOMO) energy levels in **ASMP-AP** mainly extended along the conjugate backbone while the lowest unoccupied molecular orbital (LUMO) energy levels were located almost entirely in the HBT unit. This density variation of electron cloud served rationally the driving force for ESIPT process. The theoretical band gaps of enol form and keto form were 2.9387 eV (421.90 nm) and 2.1618 eV (573.52 nm), respectively, which afforded a strong theoretical basis for subsequent experimental results.

UV–vis spectra of **ASMP-AP** exhibited an absorption maximum at 375 nm (Fig. S3) and the FL emission spectra showed blue fluorescence at around 420 nm in pure DMSO (Fig. S2). Afterwards, the investigation of the AIE feature was conducted. As the content of the poor solvent water (*f*_*w*_)increased from 60 to 80%, the molecular aggregation caused a remarkable fluorescence enhancement (~ 3.5 times compared with that in pure DMSO) at around 560 nm, wherein, however, displayed negligible fluorescence emission when the content of water was lower than 60% (Fig. S4). It clearly confirmed the AIE characteristics of **ASMP-AP,** which can be rationalized by the fact that the aggregation in AIE behavior assisted keto-emission during ESIPT. Meanwhile, it implied the large Stokes shift (~ 185 nm) which is optically favorable in living cell imaging.

**Fig. 2:**
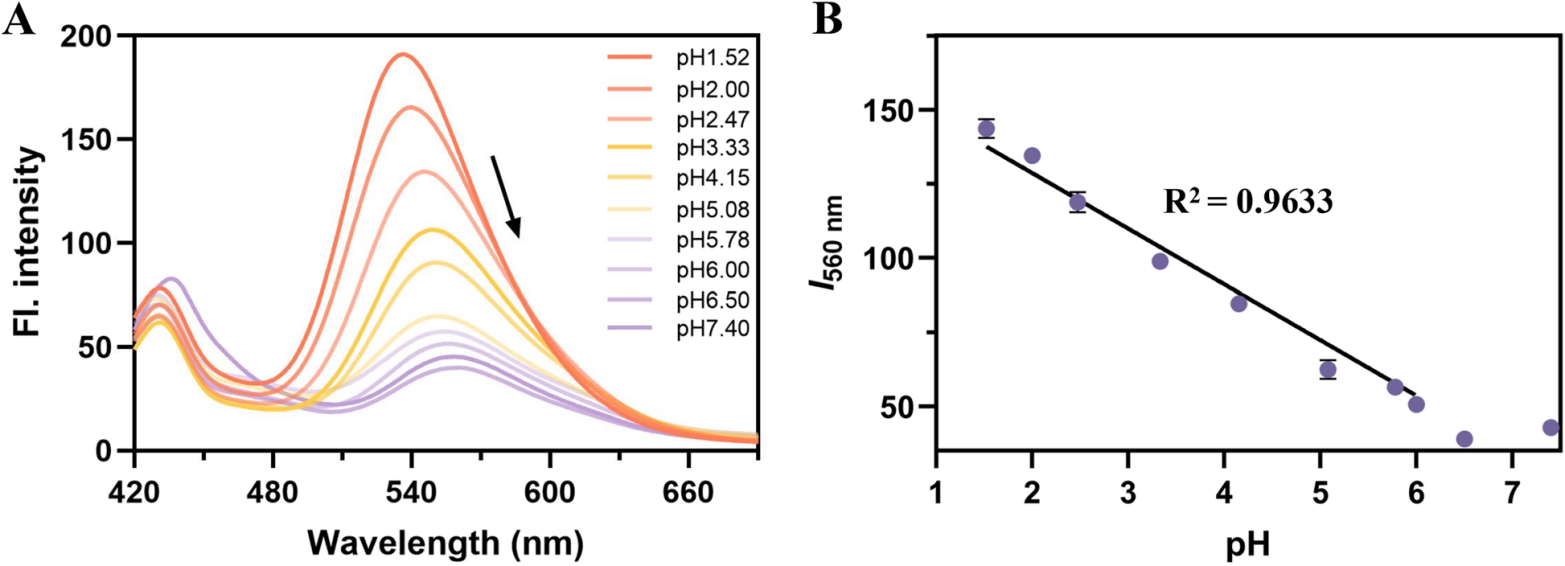
Optical response to different pH values. **A** The fluorescence emission spectra of 10 μM **ASMP-AP** in PBS buffers (10% DMSO) with varying pH condition. **B** Plots of fluorescence intensity (*I*_560nm_) vs pH

Subsequently, we explored the pH-dependent optical characteristics in PBS buffers. The fluorescence signal of **ASMP-AP** could be modulated in the range of pH from 1.52 to 6.50 (Fig. 2). In this range, with the increase of pH, the fluorescence intensity decreases accompanied with the emission bands shifts gradually from around 540 nm to 560 nm. And at physiological pH range (~ 7.4), the fluorescence signal remained almost no change. To our delight, the quantitative measurement of fluorescence intensity at 560 nm towards various pH values showed a satisfying linearity with R value high up to 0.96, which indicated the potential of **ASMP-AP** in offering dynamic lysosomal pH sensing. Noteworthy, benefiting from this linear response range, which has rarely been reported, there were opportunities to overcome the limitations encountered when detecting extremely low pH values.

This phenomenon drove us to explore the delicate mechanism. Generally, the pH sensitivity of existing probes bearing ESIPT groups is realized by harnessing the trigged fluorescence changes caused from the protonation of substrates or the effect of the hydrogen bonds. It can be broadly divided into two categories, probes with the HBT fluorophore are explained here. One is that the intramolecular hydrogen bonds are destroyed under acidic conditions, which is unfavorable for the keto emission, that cause the intensity downward tendency. It always comes at the expense of the enol emission [35]. Alternatively, combined with the perspective of AIE behavior, in a partial acid environment, the phenolic form caused via protonation possesses a lower solubility than the phenolic form under alkaline conditions, and thus more aggregation leads to enhanced fluorescence. Nevertheless, the fluorescence signal shows small pH dependence in the acidic range [36]. **ASMP-AP** is different from the prior studies. On the one hand, the stronger ketone emission at lower pH rules out the possibility of the above first type. On the other hand, the enol emission remains almost constant when pH changes. Given this, we speculated that herein the aggregation degree in aqueous solution surpassed the enol/ketone equilibrium in genal ESIPT, and became the dominant factor for affecting fluorescence. That is, the stronger aggregation as acidity increases leading to enhanced fluorescence emission. To prove this assumption, we firstly observed the pH effect on the absorptions. As displayed in the UV–vis absorption spectra at different pH (Figure S5), with the increase of acidity, the absorbance increases to a certain extent. Meanwhile, the existing level-off tails in the visible region became more significant. These observations indicated the increase of molecular aggregation and the larger particle size. To corroborate the UV–vis study, we have performed dynamic light scattering (DLS) experiments (Figure S6). As expected, it exhibited a particle size of about 615 nm at pH 2.47 while having the smaller particle size of about 295 nm with a pH reached to 5.08, which clearly demonstrated the increasing trend of aggregation as pH decreases. Consequently, these results clearly suggested that pH had an effective impact on the AIE behavior of molecules through manipulating their aggregation states. Encouragingly, we explored the specificity for pH detection in the presence of essential physiological species, including some critical metal ions, reactive oxygen species, and biological thiols, both in pH 3.3 and pH 7.4 (PBS buffer, 37 °C) (Fig. S7). It was shown that their presences caused no significant fluctuation in fluorescence intensity of probe compared to their absence. Moreover, the reversible fluorescence performance experiments by switching pH proved that **ASMP-AP** possessed good fatigue resistance (Fig. S8). These results were further evidence of the applicability for tracking pH changes in intracellular environments.

### Subcellular localization of ASMP-AP

**Fig. 3:**
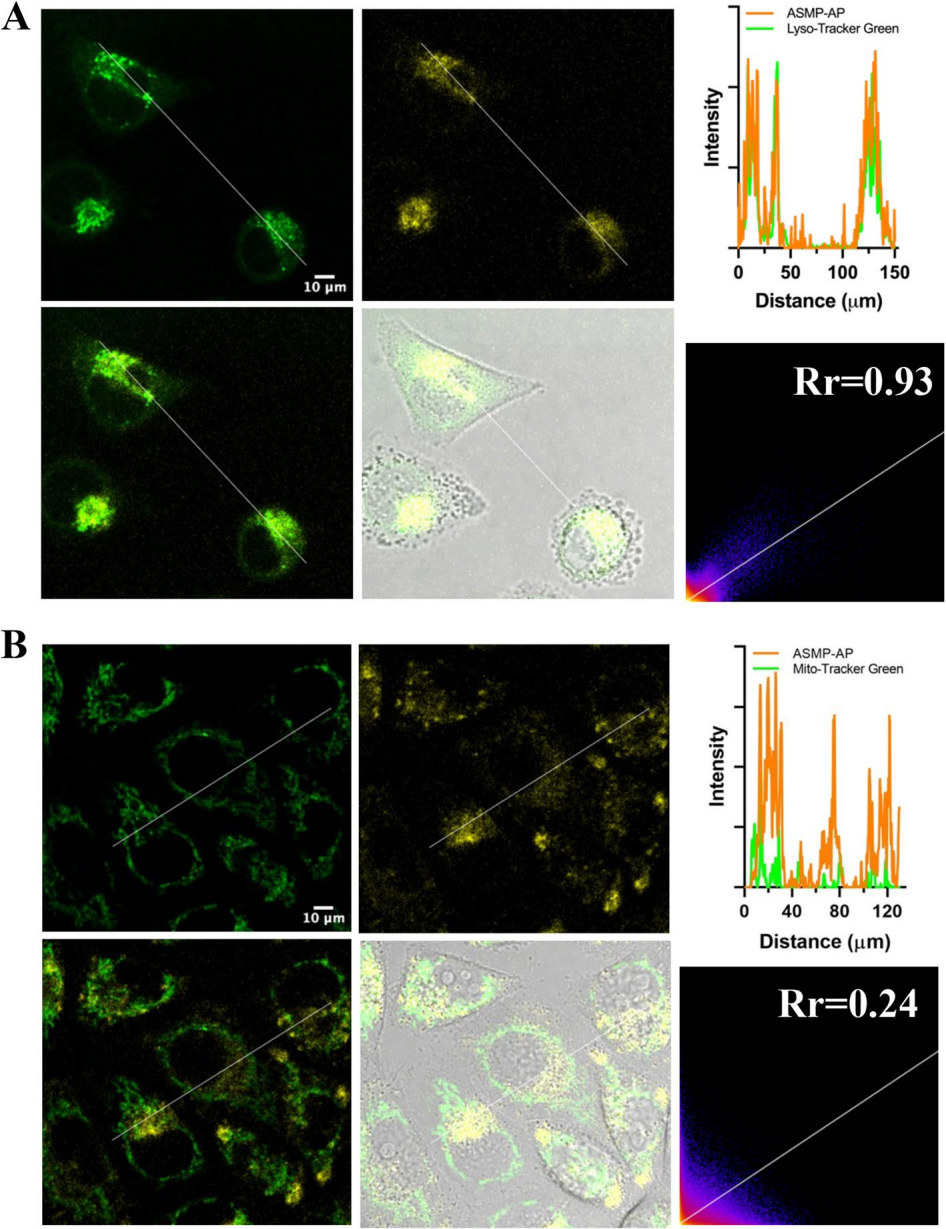
Colocalization imaging of **ASMP-AP** (10 μM, *λ*_ex_ = 405 nm, *λ*_em_ = 560–620 nm) in HeLa cells co-stained with **A**) LysoTracker green (1.0 μM, *λ*_ex_ = 488 nm, *λ*_em_ = 500–550 nm) and **B**) MitoTracker green (1.0 μM, green channel, *λ*_ex_ = 488 nm, *λ*_em_ = 500–550 nm). Cells were incubated with **ASMP-AP** at 37 °C for 30 min before the implements of other dyes. Scale bars, 10 μm

Before clarifying the performance of **ASMP-AP** at the subcellular level, the cytotoxicity of the probe was determined in HeLa cells via standard MTT assay. The cell viabilities of higher than 86% even as the probe concentration up to 40 μM after a 24-h incubation indicated good biocompatibility (Fig. S9). Next, to establish the lysosome targeting capacity, **ASMP-AP** was co-stained with Lysotracker Green and MitoTracker Green respectively in HeLa cells and imaged using confocal microscope. The spot-like yellow fluorescence of **ASMP-AP** could be clearly observed. And combining the statistically calculated high Pearson’s coefficient (0.93) in co-incubation with Lysotracker Green, it was illustrated that **ASMP-AP** enabled targeting lysosome and became more aggregated in an acidic environment (Fig. 3A). While with MitoTracker Green, the Pearson’s coefficient was shown only 0.24, further indicating the lysosomal specificity of **ASMP-AP** (Fig. 3B). Moreover, it was noticed that, benefited from the large Stokes shift, it could be seen that the fluorescence signal maintained unaffected by the backscattering of the biological matrix.

### Visualization of pH-dependent fluorescence and autophagy in live cells

**Fig. 4:**
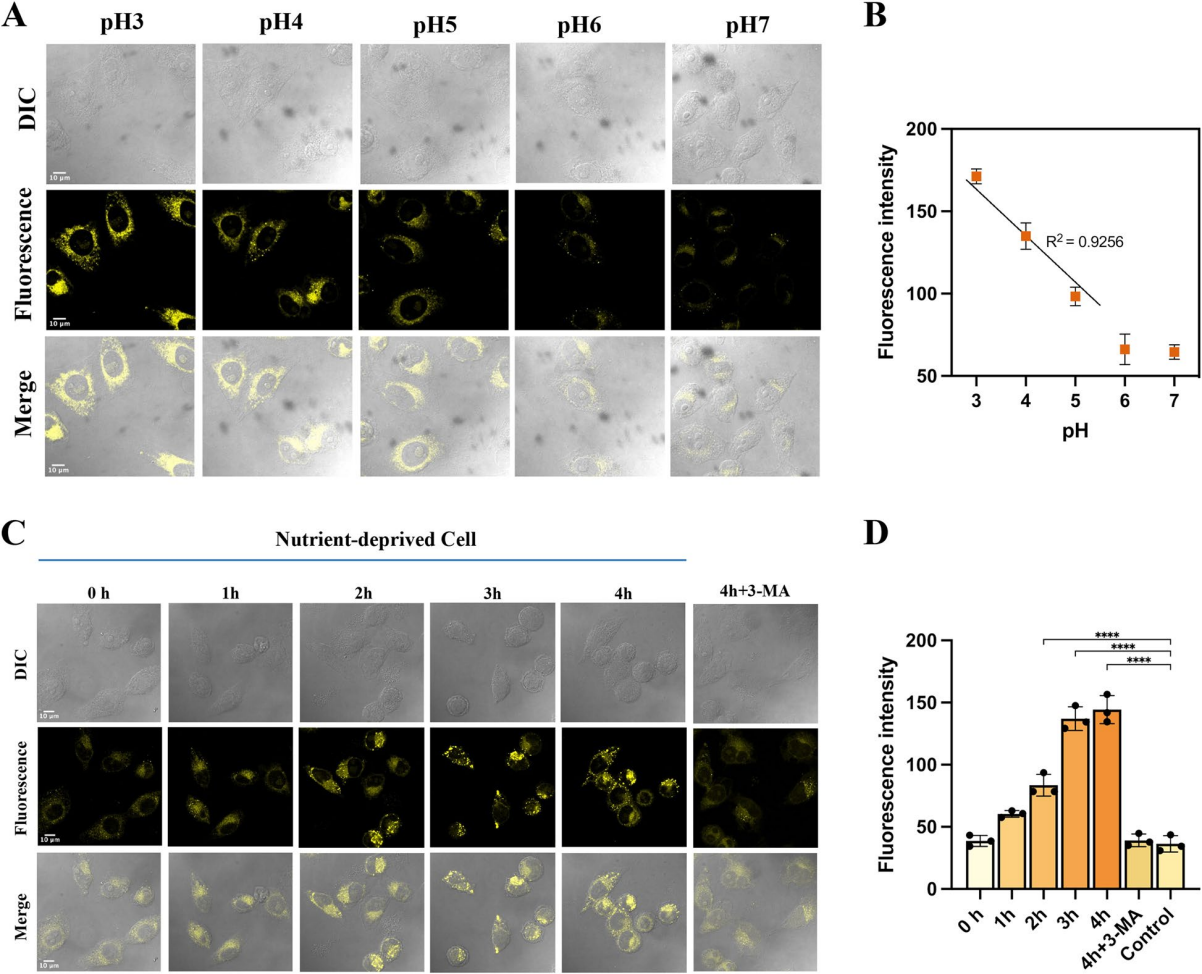
Lysosomal pH detection in HeLa cells with **ASMP-AP** (10 μM). **A**) CLSM images of cells incubated with **ASMP-AP** at 37 °C for 30 min in varied pH buffer. **B**) The plots of relative fluorescence intensities of panel A vs varied pH value. The intensities were quantified from three ROIs in each image. **C**) CLSM images of cells in Hank's balanced salt solution at different time nodes or in nutrient-rich medium. **D**) Analysis of the fluorescence intensity of panel B (*n* = 3, by ImageJ software). Statistical analyses were performed with One-way Anova. ****: *p* < 0.0001.) *λ*_ex_ = 405 nm; *λ*_em_ = 560–620 nm. Scale bars, 10 μm

**Fig. 5:**
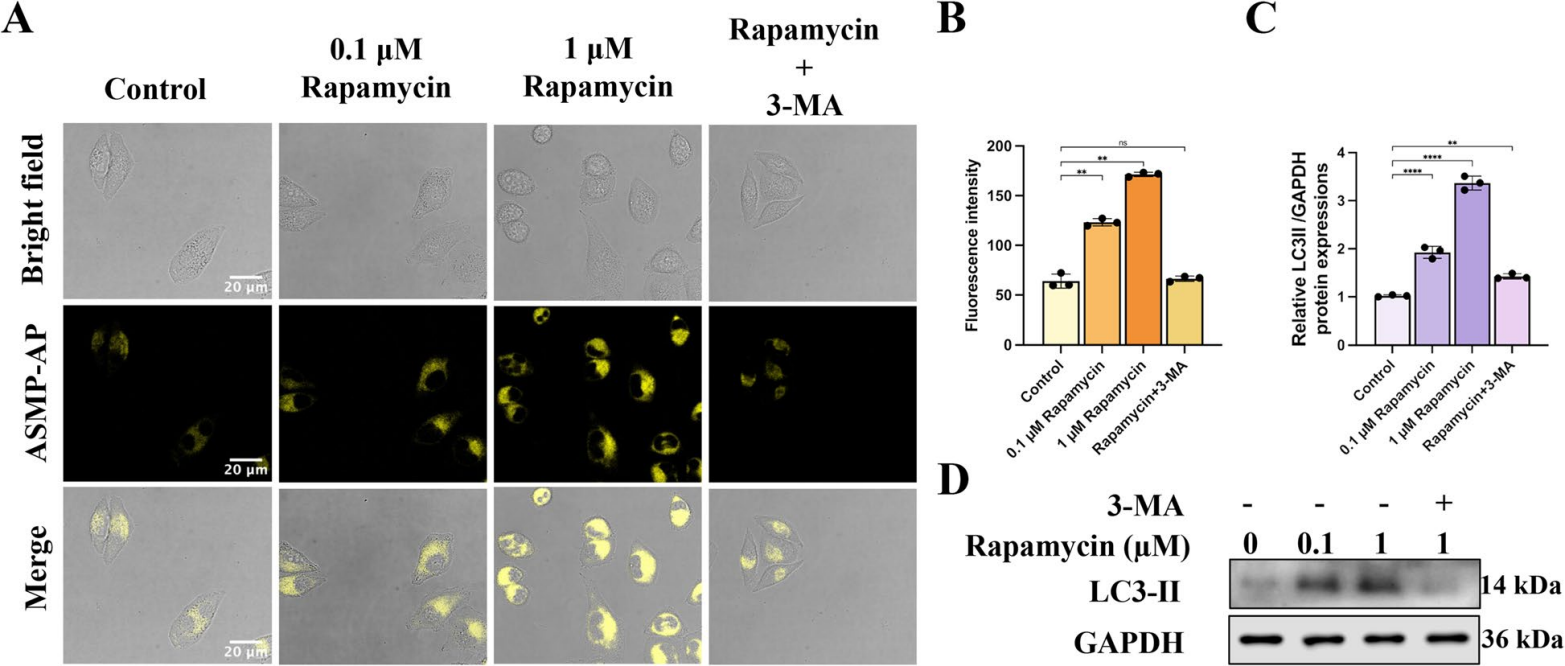
Monitoring of rapamycin-induced autophagy by **ASMP-AP**. **A** Images of HeLa cells treated with different concentrations of rapamycin, or inhibitor. *λ*_ex_ = 405 nm; *λ*_em_ = 560–620 nm. Scale bars, 20 μm. **B**, **C** Quantified fluorescence intensity of panel A (*n* = 3, by ImageJ software). **D** Western blot analysis of autophagy-related markers of HeLa cells after different treatments. Statistical analyses were performed with One-way Anova. *: *p* < 0.05, **: *p* < 0.01, ***: *p* < 0.001, and ****: *p* < 0.0001

Having established good cell permeability and lysosomal targeting ability, we sought to demonstrate the pH dependence of **ASMP-AP** in living cells. The pH of intracellular and lysosomal lumen was equilibrated using high K^+^ buffers, a standard protocol in the field [37]. As shown in Fig. 4A, at the applied pH of 3.0, the probe had bright yellow emission and exhibits a visible evident decrease in brightness as the pH increased to 7.0. Furthermore, to quantify the pH value of lysosomes, the emission intensity was plotted according to various pH values (Fig. 4B). A satisfactory linearity was obtained in the range of pH from 3.0–5.0. This built a reliable foundation for the visualization of autophagy. Next, we treated cells with serum starvation for 4 h to induce autophagy and tracked autophagy with **ASMP-AP** over starvation time. It was shown that **ASMP-AP** maintained staining during autophagy, and the yellow signal increased with the elongation of starvation time, accompanied by the increase of fluorescent spots (Fig. 4C,D). On the contrary, with the nutrient-rich medium, the yellow signals kept constant (Fig. S10). Accordingly, the expression of LC3-II (microtubule-associated protein 1 light chain 3) was monitored via Western blotting, which is an important molecule involved in the fusion of autophagosomes and lysosomes and can be degraded and recycled by enzymes together with cytoplasmic materials. It is a hallmark of autophagosome formation [38, 39]. The results revealed that the expression level of LC3-II was up-regulated continually and reached its highest at 2 h post-starvation, indicating the elevated level of autophagy. It is worth noting that the level of LC3-II appeared slightly declined after the prolonged starvation time (Fig. S11). We speculated that the occurrence of the fusion of autophagosomes with lysosomes exceeded the number of autophagosomes formed in the initial stage of autophagy, so that LC3-II was more hydrolyzed. To further validate whether such fluorescence changes originated from the occurrence of autophagy, autophagy inhibitor (3-methyladenine, 3-MA, protease inhibitors) treatment as performed, and the fluorescence signal of **ASMP-AP** was decreased dramatically. Collectively, the increase in lysosomal acidity during starvation-induced autophagy triggered an enhanced fluorescent signal thanks to the pH-dependent AIE effect of the **ASMP-AP**. In parallel, we verified the autophagy dependence of the fluorescence signal of **ASMP-AP** using different concentrations of rapamycin. Previous studies have shown that rapamycin could act on mTOR (the target of rapamycin kinase) as an autophagy activator [40]. As shown in Fig. 5A-C, the simulation with 1 μM rapamycin for 4 h to induce autophagy could elicit fluorescence intensity to increase nearly 2.7-fold, and the enhancement could be well inhibited by 3-MA. Moreover, Western blot showed an obvious increase of LC3-II expression after rapamycin treatment in a dose-dependent manner (Fig. 5D). Overall, these data suggested that **ASMP-AP** enabled visualizing the dynamic process of autophagy by interpreting the enhancement of lysosomal acidity.

### Tracking of mitophagy

**Fig. 6:**
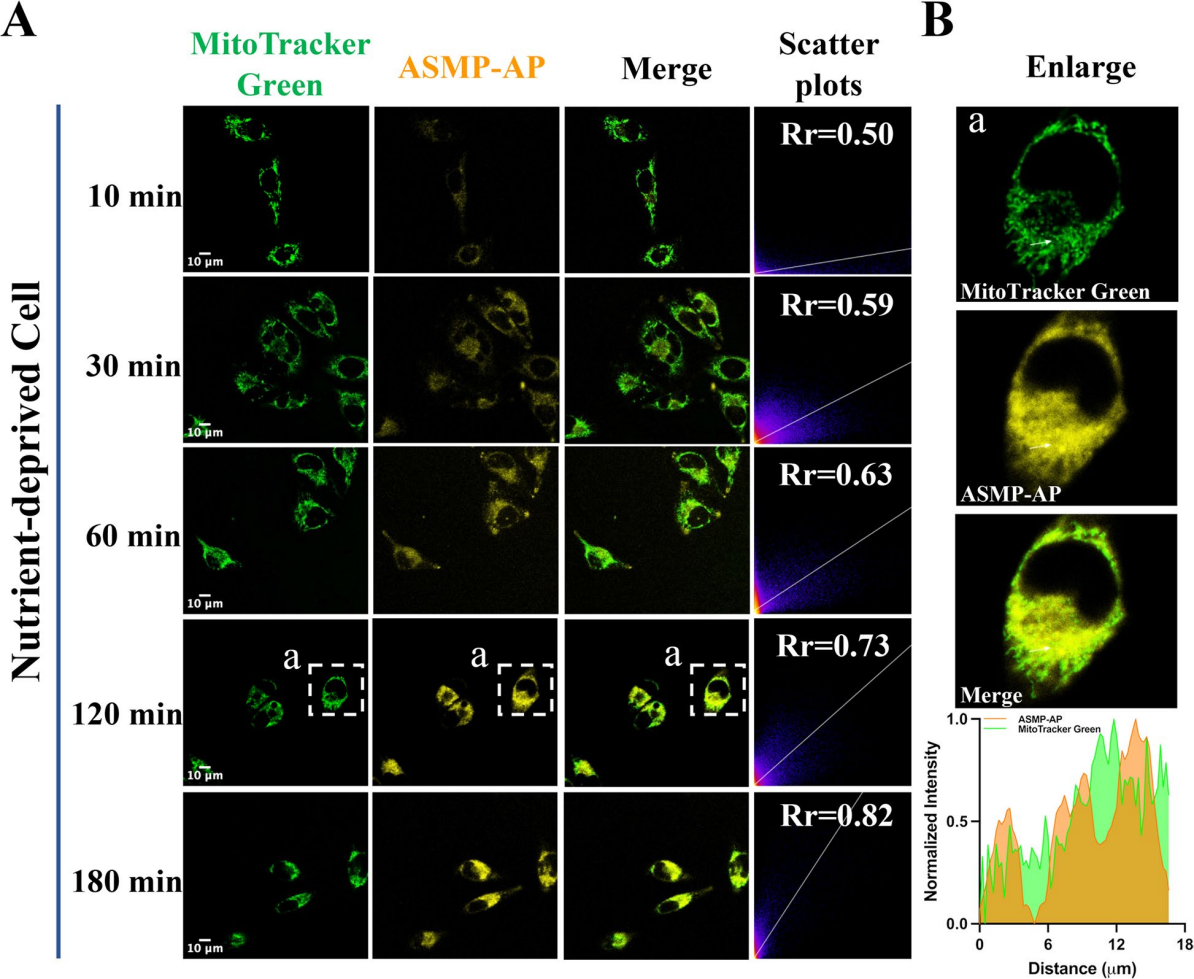
**A**) CLSM images of nutrient-deprived HeLa cells stained with **ASMP-AP** (10 μM, *λ*_ex_ = 405 nm, *λ*_em_ = 560–620 nm) and MitoTracker green (1.0 μM, *λ*_ex_ = 488 nm, *λ*_em_ = 500–550 nm) at different durations. Scale bar, 10 μm. **B**) Amplification of boxed regions in A. The plot profile presents intensities of **ASMP-AP** and MitoTracker green on the white arrows in a

As a special autophagy pathway, mitophagy can mediate lysosomes to clear damaged, aging or dysfunctional mitochondria, and its dysfunction, such as metabolic collapse and undesirable regulation, plays a vital role in the occurrence of various diseases [41–43]. It is therefore of significance to develop reliable functional tools to monitor mitophagy for investigating mechanism. To clarify the ability of **ASMP-AP** to track mitophagy, we operated **ASMP-AP** within HeLa cells in serum starvation treatment. Firstly, **ASMP-AP** was co-stained with LysoTracker green in Hank’s solution for 4 h. The excellent colocalization coefficient (0.96) proved that **ASMP-AP** possessed high affinity and long-term retention capability for lysosomes (Fig. S12). Next, we co-incubated the probe and MitoTracker green to observe the real-time colocalization in induced mitophagy. Initially, the cells showed relatively weak fluorescence in the yellow channel, giving a poor colocalization coefficient (Rr = 0.50) (Fig. 6A). As the starvation time was extended to 3 h, it was clearly observed that the yellow fluorescence of **ASMP-AP** gradually overlapped with the green fluorescence of MitoTracker green, and the colocalization coefficient gradually increased to 0.82. This indicated that, with the development of mitophagy, more mitochondria were encircled into autophagosomes and abundant fusions with lysosomes emerged. Furthermore, it was clearly manifested from the view of partial enlargement. As shown by the white arrow in Fig. 6B, the co-localization curve indicated the fact that autolysosomes containing mitochondria were generated, while the location of the separated regions implied that lysosomes and autophagosomes may be approaching. Meanwhile, as noted in the increasingly bright yellow signal, the acidity of lysosomes was enhanced during mitophagy to strengthen their functions (Fig. S13). These results supported the lysosome triggered pH sensitive **ASMP-AP** as the potential tool for tracking mitophagy.

### Imaging autophagy in ex vivo tissue

**Fig. 7:**
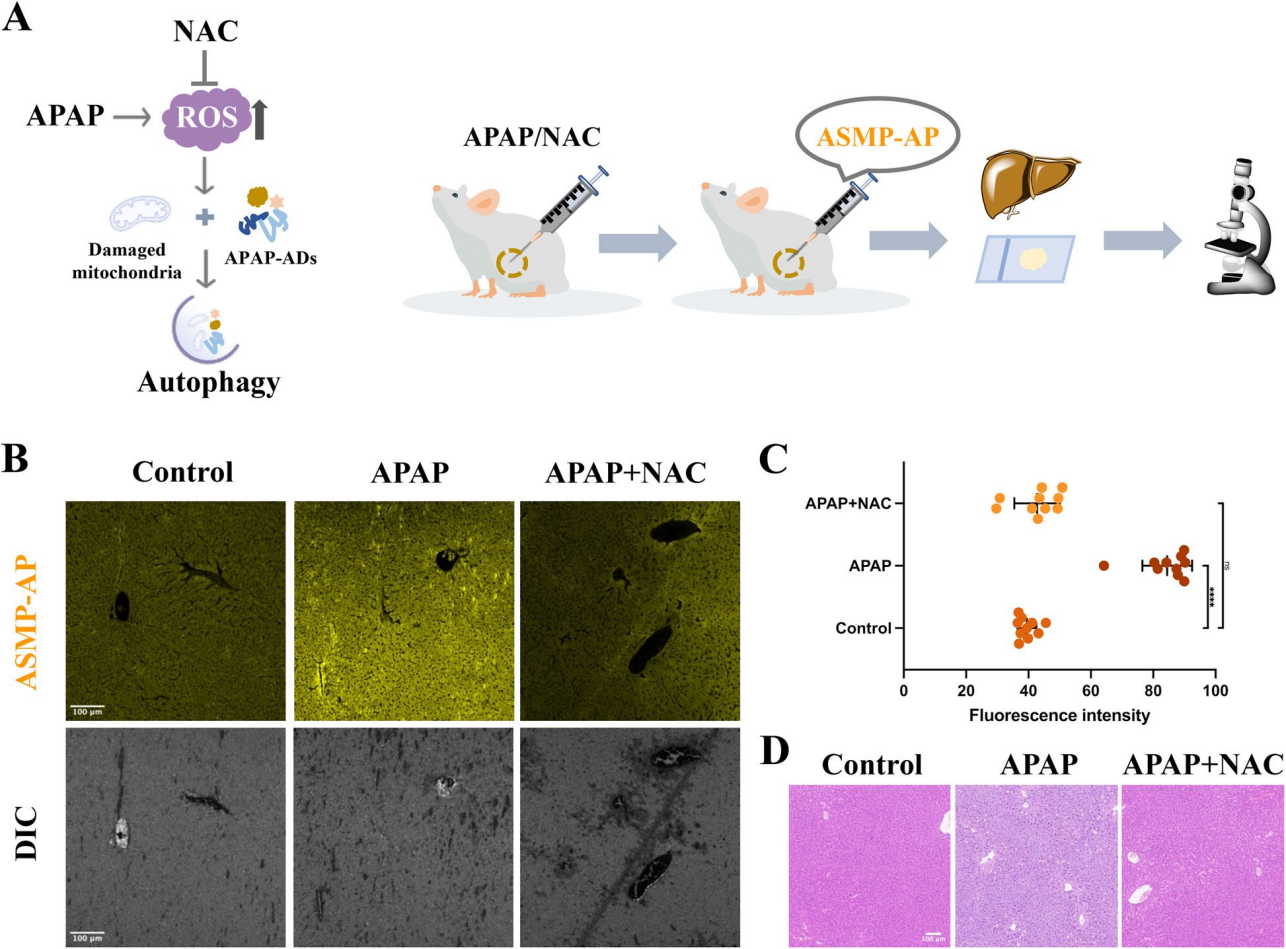
**A** Simple schematic illustration of autophagy activation in the APAP-induced liver injury model and schematic presentation of the construction of mice model and relative experiment method; **B** CLSM images of the liver tissue obtained from mice injected with **ASMP-AP**. *λ*_ex_ = 405 nm, *λ*_em_ = 560–620 nm. **C**) The quantified fluorescence intensity of **ASMP-AP** in panel B. Error bars represent standard deviation (SD) of ten ROIs. Statistical analyses were performed with One-way Anova. ****: *p* < 0.0001. **D** Representative histological analysis of the livers of mice with different treatments, saline, APAP and APAP + NAC. Scale bar, 100 µm

The outstanding performance of **ASMP-AP** prompted us to further study its potential. We expected that the bio-response of **ASMP-AP** to autophagy at the lysosomal subcellular level could also be visualized on a macroscopic scale, such as ex vivo tissue of mice. Overdose of acetaminophen (APAP) has been reported to cause acute liver failure in which induced mitochondrial oxidative stress plays a key role in pathogenesis [44]. In response to this severe cellular stress, autophagy, in an effort to maintain cellular homeostasis, is naturally activated (Fig. 7A). Accumulating evidence suggests that enhanced autophagy plays an essential role in resistance to hepatocyte injury [45]. Accordingly, the development of potential tools to track this pathophysiological process is conducive to improving the prognosis of liver injury. We applied **ASMP-AP** to the autophagy activation model in acute liver injury induced by APAP, and the histological changes of liver tissues were identified by hematoxylin–eosin staining (H&E) results (Fig. 7D). Specifically, the mice were intraperitoneally administered APAP followed by intraperitoneal (i.p.) injection of **ASMP-AP**, and then the liver tissue sections were harvested and observed by fluorescence imaging. As shown in Fig. 7B, compared with the slight fluorescence of the control group, the fluorescence signal of autophagy activation group was significantly enhanced by twofold (Fig. 7C). To verify the experimental results, 2.0 h after APAP administration, mice were administered (i.p.) with N-acetyl cysteine (NAC), which is an antioxidant that can clear the active oxygen substances and has superior capability in the hepatic protection. It has been proved that APAP-induced autophagy activation can be alleviated after NAC treatment [46]. As expected, the imaging result was in concordance with it, liver tissue slices showed obvious fluorescence inhibition compared to APAP group. These results demonstrated the good tissue permeability of **ASMP-AP** and supported the utility for reflecting autophagic activation in APAP-induced liver injury.

### ASMP-AP discerns autophagy in vivo

Complementing existing in vitro studies with in vivo experiments in animals is critical. However, the exact biologic relevance of autophagy in vivo was still obscure for years. Gratifyingly, the zebrafish model has brought dawn in the in vivo study of autophagy [47]. Not only because of their high degree of genomic conservation with the human genome, but also the advantages of optical clarity, traceability, and applicability for high-throughput screening, zebrafish models offer the possibility to study complex biological processes [48]. To assess the potential of **ASMP-AP** in tracking autophagy in vivo, we investigated the effect of rapamycin in zebrafish by co-staining with LysoTracker green. As depicted in Figure S14, 12 h-pretreatment with rapamycin elicited a distinct fluorescence signal in the yellow channel, indicating increased autophagy activity. To reinforce this conclusion, pretreatment of zebrafish with autophagy inhibitor 3-MA was performed, it was found only relatively weak fluorescence. Moreover, the signal of the **ASMP-AP** showed good overlap with that of LysoTracker green. Impressively, **ASMP-AP** could also accumulate in the eyes and brain (Figure S15). Hence, these results were suggestive of excellent tissue good cell penetration ability, excellent lysosome localization specificity, and the promising capability for monitoring autophagy in zebrafish of **ASMP-AP**.

**Fig. 8:**
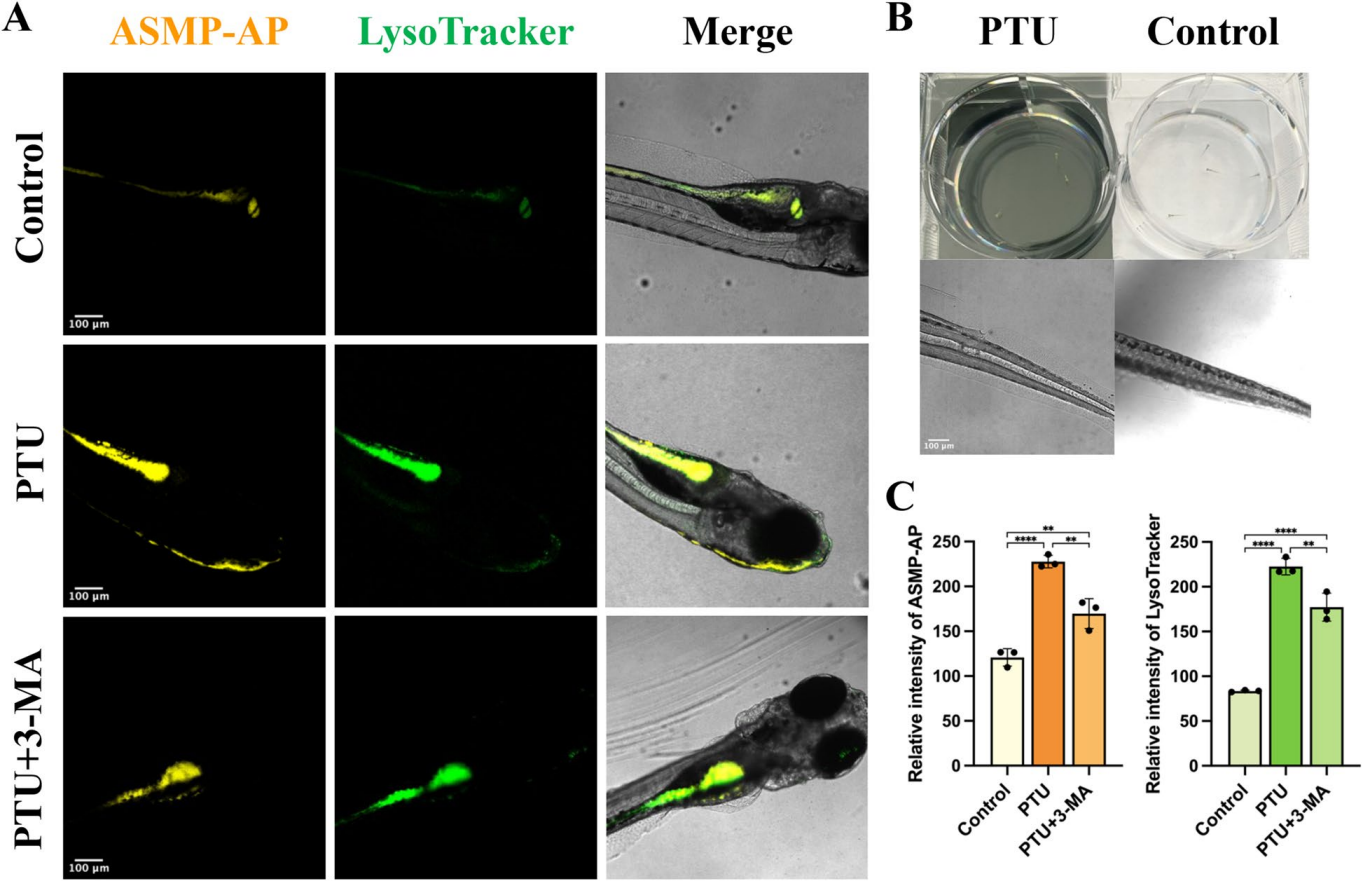
**ASMP-AP** discerns PTU-induced autophagy in zebrafish. **A** CLSM images from zebrafishes treated with PTU and stained with **ASMP-AP** (10 μM, *λ*_ex_ = 405 nm, *λ*_em_ = 560–620 nm) and LysoTracker green (1.0 μM, *λ*_ex_ = 488 nm, *λ*_em_ = 500–550 nm). **B** Representative images of inhibition of pigmentation with PTU treatment. **C** The fluorescence intensity was quantified form three independent ROIs in individual animals by ImageJ software. Statistical analyses were performed with One-way Anova. **: *p* < 0.01 and ****: *p* < 0.0001. Scale bar, 100 µm

Intriguingly, we also applied **ASMP-AP** to explore the effect of PTU, a melanin inhibitor, on autophagy by elucidating the acidification of lysosomes. In most zebrafish model studies, it has been used to inhibit melanogenesis to increase zebrafish transparency for unencumbered visualization with fluorescent probes. More than that, some physiological side effects have been reported [49, 50]. In a limited number of relevant disease models, it is necessary to avoid the bias caused by PTU. Recently, Chen et al. further revealed the induce function of PTU on autophagy in zebrafish. Therefore, it would be valuable to carry out relative investigation. Similarly, we used LysoTracker green and **ASMP-AP** to stain zebrafish pretreated with PTU. Compared with the control group, it showed a remarkable dense yellow signal, which was attributed to the increased autophagic activity interpreted by the larger lysosome shape and stronger lysosomal acidity (Fig. 8A, C). In contrast, the significant enhancement in zebrafishes pretreated with 3-MA could be suppressed. The considerable decrease of melanin of the caudal region in the bright field indicated the use of PTU (Fig. 8B). To conclude, these observations represented a proof-of-concept study to demonstrate the potential of **ASMP-AP** for in vivo visualization of the effect from PTU on autophagy.

## Discussion

Traditional tetraarylimidazole-based fluorescent scaffolds were constructed by the one-pot method which largely limits their further functionalities. To address this, we previously developed a rational strategy to construct the first asymmetric tetraarylimidazole-based probe [31]. Nevertheless, in the following characterization, we found the appending of HBT moiety on tetraarylimidazole scaffold at the 5-position not only behaved as a ESIPT occurring site, but also exhibited sensitivity towards the certain environment, in which the fluorescence would be changed. Given this, we sought out to endow asymmetric tetraarylimidazole with lysosome-targeting ability and construct a fluorescent probe for monitoring the enhanced acidity in lysosomes during autophagy.

Autophagy is a strictly regulated process which could be harnessed to develop novel cancer chemotherapies, vaccines, and other therapeutics [51, 52], it is urgently needed to develop functional molecular tools for visualizing autophagic processes so that offers a robust platform for in-depth exploration. Indeed, cytoplasmic components are transported via autophagosomes to acidic lysosomes for metabolism and recycling, which represents application niches for lysosome-targeted fluorescent probes. Moreover, the lysosomes have been reported with definite changes during autophagy, such as increased numbers, larger size, and enhanced acidification. On account of the unique characteristics, monitoring the pH various in lysosomes with fluorescent probe may provide fascinating opportunities for real-time imaging autophagy dynamics. Concurrently, AIE fluorescent materials reassuringly possess intrinsic advantages in monitoring autophagy, which is mainly embodied in that the robust fluorescence signal of aggregates enables good photobleaching tolerance and is capable of surmounting the dissipation of probes in stressed organelles [27], thereby holding potential to overcome the complexity of cellular autophagy dynamics. To the best of our knowledge, however, only Leung’s group employed a AIE probe to detecting lysosomal pH in autophagy related research where the working principle utilize the influence of hydrogen bonds and the acidity responses exhibit “turn-off” behavior. In addition, there is no investigation of monitoring autophagy on histological level or in vivo in their work.

In this study, we de novo design a novel pH sensitive AIE fluorescent probe **ASMP-AP** with lysosomal targeting for autophagy visualization. The morpholine group is elaborately deployed at the beginning of synthesis to confer lysosomal specificity, which seems infeasible by the previous one-pot method. The investigation into the spectroscopic properties and the detailed mechanism demonstrated that the higher aggregation degree of probe molecules under more acidic condition is responsible for the fluorescence enhancement. The acidity sensitivity and lysosome-targeting ability were validated in the confocal fluorescence imaging of living cells. In particular, the higher aggregation degree of probe molecules under more acidic condition not only provide significant fluorescence enhancement, but exhibited high on-site retaining. After that, the capability of **ASMP-AP** for tracking autophagy was verified in living cells under starvation stress as well as drug treatments. Furthermore, since the molecular mechanism of APAP-involved autophagy remains unclear, the translatable knowledge obtained from mouse model here will help explore how to make use of autophagy indicators to develop new diagnostic and therapeutic approaches for drug-induced liver injury. Additionally, for analyzing complex dynamic autophagy processes in intact, living organisms, zebrafish model emerges distinctive promise over other animals model [47]. Of note, Chen’s group has reported that PTU, a drug widely used in increasing transparency of zebrafish, could induce autophagy elevated. Given this, as a proof of concept, we continue to validate the inducible effect of PTU through employing wild-type zebrafish without any other drug pretreatments. It represents the first instance of novel small-molecule fluorescent probe in PTU effect study on autophagy, offering a simplified yet reliable tool for future relevant physiological mechanism researches. Collectively, we reported the first instance of a novel pH-sensitive AIE probe for autophagy visualization in cells, tissues and in vivo. More importantly, the elaboration of de novo design strategy adopted herein to generate functional AIE fluorophores can exert the various subcellular organelles or biomolecules targeting.

Nevertheless, **ASMP-AP** could only achieve specifical labeling of lysosome. Dual-labeling probes capable of labeling lysosomes and other subcellular organelles simultaneously will have potential to describe more detailed intermediates in autophagy process, and future research can focus on this.

## Conclusion

In summary, we implemented a de novo design strategy to fabricate a novel pH dependent AIE probe that exhibits good adaptability throughout the synthetic route. It enables generating an asymmetric tetraarylimidazole fluorophores with inherent lysosomal targeting and followed by appending with HBT moiety to elongate the Stokes shift. Although several other ESIPT probes characterized with AIE have been applied for detecting pH during autophagy, the shrewd coordination of AIE and ESIPT reported here brings several unique advantages: the uniqueness of the pH-manipulated aggregation states; the feasibility of extremely low pH detection; the ability to overcome the impact of acidity on intramolecular hydrogen bonding. Moreover, benefited from the intrinsic AIE properties, **ASMP-AP** achieves long-term retention capability. We shed light on how **ASMP-AP** was applied for the monitoring autophagy activity in vitro and in vivo by providing lysosomal pH fluctuations information. We corroborated the exceptional performance of **ASMP-AP** in visualizing autophagy by starvation or drug treatment, and that signal changes could be specifically blocked by autophagy inhibitor. To be highlighted, we also demonstrated the capability of **ASMP-AP** to monitor autophagy in ex vivo liver tissue and zebrafish, especially the elevated autophagic activity caused by PTU could be interpreted through the increase of lysosome number and acidity. This is the first analysis of PTU-induced autophagy using novel small-molecule fluorescent probes to track pH varies in vertebrates. Thus, we anticipate **ASMP-AP** may be suitable for autophagy-related diagnostic imaging. This design strategy based on asymmetric tetraarylimidazole fluorophores also allows expansion to other organelles and functions, and related efforts are in progress.

### Experimental section

## Materials and apparatus

Aniline, 4-morpholinobenzonitrile, phenylacetyl chloride, 2-aminothiophenol and other chemical compounds were purchased from Aladdin, Bidepharm or Energy chemical. LysoTracker Green and MitoTracker Green were purchased from Beyotime. The general biochemical agents such as Dulbecco’s modified eagle medium (DMEM), fetal bovine serum (FBS) and 3-(4,5-dimethylthiazol-2-yl)-2,5-diphenyltetrazolium bromide (MTT) were purchased from Sigma-Aldrich. Bruker AVANCE-400 and 500 MHz NMR instruments were employed for ^1^H-NMR and ^13^C-NMR spectrum. Exact mass of compounds was identified by electrospray ionization mass spectrum (ESI–MS). The UV–vis spectra and fluorescence emission spectra were recorded on Shimadzu UV-2600 absorption spectrometer and HITACHI F-7100 fluorescence spectrophotometer at 25 °C, respectively. Dynamic light scattering (DLS) study was recorded on Nano ZS90. The absorbance was recorded with microplate reader SpectraMax M2 in the cytotoxicity experiment. The confocal laser microscopy imaging was performed on Leica TCS SP8 confocal laser-scanning microscope.

### Synthesis of **ASMP-AP**

**ASMP-AP** was synthesized in six steps as depicted in Scheme S1. The initial cyclization reaction was carried out by our previously reported method to produce asymmetric tetraarylimidazole compound **2**. To the solution of compound **4** and 2-aminothiophenol in ethanol was added hydrogen peroxide and hydrochloric acid, and then allowed the reaction mixture stirred at 25 °C for two hours. The reaction solution was poured into water and extracted with CH_2_Cl_2_, and the organic phase was dried over anhydrous magnesium sulfate. Following the evaporation of the solvent under reduced pressure, the crude product was purified by silica gel column chromatography with eluent ethyl acetate/petroleum ether (1: 8, v/v) to obtain **ASMP-AP** as a brownish yellow solid (28 mg, yield 51.3%). ^1^H NMR (500 MHz, DMSO-*d6*) *δ* 11.59 (s, 1H), 8.40 (s, 1H), 8.13 (d, *J* = 7.9 Hz, 1H), 8.05 (d, *J* = 8.1 Hz, 1H), 7.57 – 7.53 (m, 2H), 7.48 – 7.43 (m, 2H), 7.40 (dd,* J* = 8.6, 2.2 Hz, 2H), 7.37 – 7.34 (m, 3H), 7.33 (d, *J* = 2.9 Hz, 2H), 7.28 (d, *J* = 3.6 Hz, 1H), 7.27 (d, *J* = 2.5 Hz, 2H), 7.25 (d, *J* = 3.9 Hz, 1H), 6.94 (d, *J* = 8.6 Hz, 1H), 6.85 (d, *J* = 8.9 Hz, 2H), 3.71 (t, *J* = 4.8 Hz, 4H), 3.15 – 3.11 (m, 4H). ^13^C NMR (125 MHz, DMSO-*d6*) *δ* 166.00, 155.46, 153.81, 151.91, 151.03, 146.75, 140.72, 139.56, 137.48, 136.29, 134.60, 131.62, 130.87, 130.46, 129.53, 129.28, 129.01, 128.77, 126.81, 125.65, 124.58, 123.62, 123.18, 122.58, 121.11, 118.35, 117.26, 116.05, 114.38, 66.47, 55.37. HRMS: m/z calcd for C_38_H_30_N_4_O_2_S ([M + H]^+^) 607.2168, found 607.2135.

### Optical response of **ASMP-AP** to pH

Stock solutions (4.0 mM) of **ASMP-AP** were prepared in DMSO. The accurate various pH values of PBS buffer were determined by PHS-3C digital pH meter. The spectroscopic measurements of pH response were tested in PBS buffer (containing 10% DMSO) with a final concentration of 10 μM of the probe. Finally, the measurements were performed on F-7100 at 25 °C.

### Cells culture for imaging and cytotoxicity assay

HeLa cells were cultured in DMEM supplemented with 10% FBS and 1% Antibiotic–Antimycotic on 35-mm glass-bottom petri dishes, and under the humidified atmosphere of 5% CO_2_ at 37 °C. For living cell imaging, the cells were incubated with 10 μM **ASMP-AP** for different times. Leica TCS SP8 Confocal Microscope was adopted to capture images with using a 64 × oil immersion objective lens. For cytotoxicity assay, the HeLa cells were firstly seeded into 96-well plates at 4 ~ 5 × 10^4^ cells/well for 24 h and treated with various concentrations of **ASMP-AP** for 24 h. Then, to each well was added 10 μL MTT (5 mg/mL) and kept cells incubated for 4 h. Eventually, following the careful removal of the medium, 100 μL DMSO was added to dissolve the crystals. The absorbance at 490 nm of each well was detected by Tecan Infinite M200 monochromator-based multifunction microplate reader.

### Colocalization experiments

HeLa cells were simultaneously incubated with **ASMP-AP** (10 μM) and LysoTracker Green DND-99 (1.0 μM) or MitoTracker Green (1.0 μM) in Hank's balanced salt solution for 0.5 h at 37 °C. Washed with PBS for three times before imaging. Linear ROIs were drawn by ImageJ software.

## Tracking of autophagy and mitophagy

For monitoring lysosomal dynamics in autophagy, HeLa cells were pretreated with **ASMP-AP** (10 μM) and accepted different stimuli, starvation (Hank’s balanced salt solution) for 0–4 h, rapamycin (0.1 and 1.0 μM) treatments for 4 h. For inhibition studies, cells were treated with 3-MA (300 μM) and the above-mentioned stimulus simultaneously. For monitoring mitophagy, after labeling the cells with **ASMP-AP** (10 μM) and MitoTracker (1.0 μM), the cells were incubated on the condition to induce mitophagy. CLSM images were captured at different time intervals. The colocalization coefficients were calculated by ImageJ software.

### Confocal microscope imaging of ex vivo mice tissue

All animal experiments were carried out in accordance with protocols approved by the Committee of the Use of Laboratory and Research Animals (CULATR). The 5-week-old BALB/c type mice were split into three groups. For APAP group, mice were pretreated with 500 mg kg^−1^ APAP (300 μL) for 10 h, and then **ASMP-AP** (300 μM, 100 μL) was injected intraperitoneally for 2 h. For NAC group, mice were administered intraperitoneally with 1.8 mmol kg^−1^ NAC (100 μL) at 2 h post APAP administration, and **ASMP-AP** was injected after 8 h. After treatments, mice were sacrificed, and the liver was excised followed by washing with 0.9% saline. Finally, the liver tissue was embedded in paraffin. The confocal microscope images of liver tissue slides were acquired with excitation 405 nm and emission 560 nm. For histological analysis, the liver tissue was fixed in 4% formalin solution followed by staining with hematoxylin and eosin (H&E).

## In vivo imaging of autophagy in zebrafish

Wild-type zebrafish embryos purchased from Nanjing Eze-Rinka company Co., Ltd were transferred into 6-wells plates in E3 medium and cultured at a constant temperature of 28 °C. For autophagy imaging, 6-day old zebrafish were incubated with rapamycin (10 μM) or 0.003% PTU (200 μM or 1X) for 12 h. And 5 mM 3-MA was applied for inhibition studies. **ASMP-AP** and LysoTracker green were diluted in E3 medium to the final concentration of 15 μM and 1 μM, respectively. After 15-min incubation, the zebrafish larvae were transferred into 35 mm glass-bottom confocal dish and rinsed three times with E3 medium. Confocal images were recorded on Leica TCS SP8 Confocal Microscope with 10 × objective lenses.

## Supplementary Information

**Additional file 1: Scheme S1.** Synthetic routine of the ASMP-AP. **Figure S1.** The constructed potential energy scan surface of both S0 and S1 states of ASMP-AP as a function of O7-H49. **Figure S2.** Fluorescence spectra of ASMP-AP (10 µM) in different solvents. λex = 375 nm. **Figure S3.** Absorption spectra of ASMP-AP (10µM) in DMSO and in aqueous solution (1% DMSO). **Figure S4.** AIE properties of ASMP-AP. a) Fluorescence spectra of ASMP-AP (10 µM) in DMSO/water mixtures with different volume fraction of water (fw). b) Fluorescence emission at 560 nm of ASMP-AP (10 µM) in DMSO/water mixed solvent. λex = 375 nm. **Figure S5.** Absorption spectra of ASMP-AP in aqueous solution (10% DMSO) with different pH. **Figure S6.** DLS spectra of ASMP-AP (10 µM) in PBS buffer at different pH values. **Figure S7.** Fluorescence responses of ASMP-AP (10 µM) to pH 3.33 and 7.40 in the absence and presence of possible interfering substances at 37 °C, respectively. Such as metal ions (100 µM), essential thiols (1 mM), H2O2 (100 µM). **Figure S8.** Emission maximum versus switching cycles between pH 3.33 and pH 7.4. **Figure S9.** Cytotoxicity of ASMP-AP to Hela cells determined by MTT assay. The data are based on the average and show the standard deviation (*n* = 3). **Figure S10.** CLSM images of cells in in nutrient-rich medium at different time nodes. λex = 405 nm; λem = 560-620 nm. Scale bars, 10 μm. **Figure S11.** Autophagy-related protein expression and statistical analysis under starvation conditions. **Figure S12.** Lysosome-targeting properties of ASMP-AP in HeLa cells after 4 h-starvation treatment. Colocalization images stained with LysoTracker Green (1.0 μM, green channel, λex = 488 nm, λem = 500-550 nm) and ASMP-AP (10 μM, yellow channel, λex = 405 nm, λem = 560-620 nm), and the correlation of ASMP-AP and LysoTracker Green intensities as well as the intensity profiles within the ROI. Scale bars, 10 µm. **Figure S13.** Analysis of the fluorescence intensity of ASMP-AP channel in panel Figure 6A (*n* = 6, by ImageJ software). Statistical analyses were performed with One-way Anova. *: *p* < 0.05, ****: *p* < 0.0001. **Figure S14.** Confocal fluorescence images of zebrafish larvae in rapamycin induced autophagy. A) Followed by the pretreatment with rapamycin for 12 h at 28 ℃, zebrafish larvae were stained with ASMP-AP (10 μM, λex = 405 nm, λem = 560-620 nm) and LysoTracker green (1.0 μM, λex = 488 nm, λem = 500-550 nm). Statistical analyses were performed with One-way Anova. *: *p* < 0.05, **: *p* < 0.01, ***: *p* < 0.001, and ****: *p* < 0.0001. B) Colocalization relationship of ASMP-AP with LysoTracker green. Scale bars, 100 µm. **Figure S15.** Confocal fluorescence images of different positions of zebrafish larvae (eyes and midbrain) with rapamycin treatment. ASMP-AP (10 μM, λex = 405 nm, λem = 560-620 nm). Scale bars, 100 µm. **Table S1.** Comparison of properties of various pH-sensitive AIE probes. **Figure S16.** 1H NMR spectra (DMSO-d6)) of compound 1. **Figure S17.** 13C NMR spectra (DMSO-d6)) of compound 1. **Figure S18.** HRMS spectrum of compound 1. **Figure S19.** 1H-NMR spectra (DMSO-d6) of compound 2. **Figure S20.** 13C NMR spectra (DMSO-d6) of compound 2. **Figure S21.** HRMS spectrum of compound 2. **Figure S22.** 1H-NMR spectra (DMSO-d6) of compound 3. **Figure S23.** HRMS spectrum of compound 3. **Figure S24.** 1H NMR spectra (DMSO-d6) of compound 4. **Figure S25.** 13C NMR spectra (DMSO-d6) of compound 4. **Figure S26.** HRMS spectrum of compound 4. **Figure S27.** 1H NMR spectra (DMSO-d6) of ASMP-AP. Figure S28. 13C NMR spectra (DMSO- d6) of ASMP-AP. Figure S29. HRMS spectrum of compound ASMP-AP.

## Data Availability

The datasets used and/or analyzed in this study are available from the corresponding author on reasonable request.

## Abbreviations

ACQ: Aggregation-caused quenching
AIE: Aggregation-induced emission
PTU: 1-Phenyl-2-thiourea
ESIPT: Excited-state intramolecular proton transfer
RIM: Restriction of intramolecular motion
PET: Photoinduced electron transfer
ICT: Internal charge transfer
PES: Potential energy surface
FMOs: Frontier molecular orbitals
HOMO: Highest occupied molecular orbital
LUMO: Lowest unoccupied molecular orbital
LC3-II: Microtubule-associated protein 1 light chain 3
3-MA: 3-Methyladenine
APAP: Acetaminophen
H&E: Hematoxylin–eosin staining
NAC: N-acetyl cysteine
