# Supplementary material for: De novo design of a novel AIE fluorescent probe tailored to autophagy visualization via pH manipulation
Source: Biomater Res. 2023 Mar 13;27:20. doi: 10.1186/s40824-023-00359-w (PMC10012510; doi:10.1186/s40824-023-00359-w)
Supplement: Supplementary file 1 — Additional file 1: Scheme S1. Synthetic routine of the ASMP-AP. Figure S1. The constructed potential energy scan surface of both S0 and S1 states of ASMP-AP as a function of O7-H49. Figure S2. Fluorescence spectra of ASMP-AP (10 µM) in different solvents. λex = 375 nm. Figure S3. Absorption spectra of ASMP-AP (10µM) in DMSO and in aqueous solution (1% DMSO). Figure S4. AIE properties of ASMP-AP. a) Fluorescence spectra of ASMP-AP (10 µM) in DMSO/water mixtures with different volume fraction of water (fw). b) Fluorescence emission at 560 nm of ASMP-AP (10 µM) in DMSO/water mixed solvent. λex = 375 nm. Figure S5. Absorption spectra of ASMP-AP in aqueous solution (10% DMSO) with different pH. Figure S6. DLS spectra of ASMP-AP (10 µM) in PBS buffer at different pH values. Figure S7. Fluorescence responses of ASMP-AP (10 µM) to pH 3.33 and 7.40 in the absence and presence of possible interfering substances at 37 °C, respectively. Such as metal ions (100 µM), essential thiols (1 mM), H2O2 (100 µM). Figure S8. Emission maximum versus switching cycles between pH 3.33 and pH 7.4. Figure S9. Cytotoxicity of ASMP-AP to Hela cells determined by MTT assay. The data are based on the average and show the standard deviation (n = 3). Figure S10. CLSM images of cells in in nutrient-rich medium at different time nodes. λex = 405 nm; λem = 560-620 nm. Scale bars, 10 μm. Figure S11. Autophagy-related protein expression and statistical analysis under starvation conditions. Figure S12. Lysosome-targeting properties of ASMP-AP in HeLa cells after 4 h-starvation treatment. Colocalization images stained with LysoTracker Green (1.0 μM, green channel, λex = 488 nm, λem = 500-550 nm) and ASMP-AP (10 μM, yellow channel, λex = 405 nm, λem = 560-620 nm), and the correlation of ASMP-AP and LysoTracker Green intensities as well as the intensity profiles within the ROI. Scale bars, 10 µm. Figure S13. Analysis of the fluorescence intensity of ASMP-AP channel in panel Figure 6A (n = 6, by ImageJ softwa [file 40824_2023_359_MOESM1_ESM.docx]

Supporting Information

***De novo* design of a novel AIE probe tailored to autophagy visualization via pH manipulation**

*Xueyan Huang,**^a,b,‡^ Fei Chen,^a,b^*,*^‡^ Yeshuo Ma, ^a,b^ Fan Zheng, ^a,b^ Yanpeng Fang, ^a,b^ Bin Feng, ^a,b^ Shuai Huang, ^a,b^ Hongliang Zeng, ^c^ Wenbin Zeng*^a,b^*

^a^ Xiangya School of Pharmaceutical Sciences, Central South University, Changsha, 410013, PR China.

^b^ Hunan Key Laboratory of Diagnostic and Therapeutic Drug Research for Chronic Diseases, Changsha, China

^c^ Hunan Academic of Chinese Medicine, Inst Chinese Mat Med, Changsha, PR China.

* Corresponding Author: Prof Dr W Zeng, E-mail address: wbzeng@hotmail.com. Tel/Fax: 0086-731-82650459. Dr H Zeng, E-mail address: zenghl155@163.com.

**Contents**

[General methods S3](#_Toc119413006)

[Experimental section S4](#_Toc119413010)

[Theoretical ESIPT analysis S7](#_Toc119413011)

[Optical properties and biological studies S8](#_Toc119413012)

[Characterization of compounds S14](#_Toc119413013)

# General methods

## Theoretical methods

The quantum chemical calculations were performed on the Gaussian 16 program. The geometric structure of excited species was optimized by time dependent density functional theory (TD-DFT) based on PBE0/def2-SVP level. The molecular orbital (MO) plots and energy levels were calculated with the same level of theory. And the molecular orbitals were visualized using VMD.[[1](#_ENREF_1)] To simulate the experimental solvent environment, the integral equation formalism (IEF) variant of the polarizable continuum model (PCM) calculation was employed with DMSO as the solvent to consider the solvation effect. To investigate the ESIPT process mechanism, the potential energy curves were scanned in the ground state (S_0_) and the first excited state (S_1_) as a function of the bond length of O_7_-H_49_ based on PBE0/def2-SVP level.

## pH detection in living cells

HeLa cells were incubated with **ASMP-AP** (10 μM) for 30 min at 37 ℃, then washed with PBS for three times. The medium was replaced with high-K^+^ buffer (30 mM NaCl, 120 mM KCl, 1.0 mM CaCl_2_, 0.5 mM MgSO_4_, 1.0 mM NaH_2_PO_4_, 20 mM NaOAc, 5.0 mM glucose, 20 mM HEPES) at different pH values (3.0-7.0). After 5 min incubation, the images were recorded on the confocal microscope.

## Western blotting

HeLa cells after starvation or drug treatment were collected at the corresponding time points and lysed in RIPA lysis buffer. Then centrifuged at 15000 r.p.m. for 10 min and collected the supernatant. The samples were separated by electrophoresis (15% SDS-PAGE), and then transferred to the polyvinylidene-difluoride (Millipore) membrane for electroblotting for 90 min. The membrane was blocked with 5% skimmed milk at room temperature for 1.5 h, incubated with anti-LC3 antibodies and anti-GAPDH antibodies at 4 ° C overnight. The immune signal was detected after 1 h incubation with HRP-conjugated secondary antibody. For zebrafish larvae, RIPA lysis buffer was used to extract protein. Samples were centrifuged the sample at 12000🞨*g* for 5 min and heated at 95 ° C for 5 min before electrophoresis. Then follow the above ascribed operation.

# Experimental section

**Scheme S1.** Synthetic routine of the **ASMP-AP**.

**Synthesis of benzyl 4-methoxyphenyl ketone**

Benzyl 4-methoxyphenyl ketone was synthesized following our previous procedures. To the solution of phenylacetyl chloride (620 mg, 4 mmol) in 1,2-dichloroethane (20 mL) was added aluminum trichloride (802.8 mg, 6 mmol) at 0 ℃. Then the anisole (432 mg, 4 mmol) was added dropwise at a maintained temperature of 0- 5 ℃. The mixture was warmed to room temperature and stirred for 2 h. Then the resulting mixture was poured into a mixture of ice water and 36% hydrochloric acid. The aqueous solution was extracted with CH_2_Cl_2_ and washed with brine. The organic layer was dried over anhydrous sodium sulfate and concentrated by vacuum-rotary evaporation procedure. Finally, the residue was purified by column chromatography with ethyl acetate/petroleum ether (1: 60, v/v) as eluent to obtain **1** as a white solid (464 mg, 51.3%). ^1^H NMR (500 MHz, CH_3_OD) δ 8.04 – 8.00 (m, 2H), 7.27 (ddd, *J* = 14.5, 7.9, 4.1 Hz, 4H), 7.22 – 7.19 (m, 1H), 6.98 (d, *J* = 9.0 Hz, 2H), 4.26 (s, 2H), 3.85 (s, 3H). HRMS: m/z calcd for C_15_H_15_O_2_ ([M + H]^+^) 227.1072, found 227.1064.

**Synthesis of compound 1**

To the solution of aniline (298.0 mg, 3.2 mmol) in dry tetrahydrofuran was added sodium hexamethyldisilazide (1.5 mL, 1 M solution in THF) dropwise at 0 ℃ under nitrogen protection. After stirring for 20 min, a solution of benzonitrile (600.0 mg, 3.2 mmol) in THF was slowly added. Then allowed the reaction stirred overnight at room temperature. After that, the mixture was poured into ice water (400 mL) and extracted with dichloromethane. The collected organic solution was dried over anhydrous magnesium sulfate and concentrated by reduced pressure. The concentrate was recrystallized in a system of dichloromethane and petroleum ether to obtain **1** as a brown solid (726.0 mg, 80.6%). ^1^H-NMR (400 MHz, DMSO-*d_6_*) δ 7.87 (d, J = 8.6 Hz, 2H), 7.30 (t, J = 7.7 Hz, 2H), 6.96 (d, J = 8.9 Hz, 3H), 6.85 (d, J = 7.9 Hz, 2H), 6.11 (s, 2H), 3.79 – 3.72 (m, 4H), 3.23 – 3.16 (m, 4H). ^13^C-NMR (101 MHz, DMSO) δ 152.68, 151.24, 129.59, 128.52, 126.24, 122.25, 121.96, 114.01, 66.46, 48.20. HRMS: m/z calcd for C_17_H_19_N_3_O ([M + H]^+^) 282.1606, found 282.1596.

**Synthesis of compound 2**

To the solution of benzyl 4-methoxyphenyl ketone (217.0 mg, 0.96 mmol) in acetonitrile (10 mL) was added the carbon tetrabromide (382.0 mg, 1.15 mmol) dissolved in acetonitrile slowly. Compound 1 (539.0 mg, 1.92 mmol) dissolved in acetonitrile was slowly added into the above mixture. Then the reaction was stirred overnight at 70 ℃. The solvent was evaporated under reduced pressure and the residue was purified by silica column chromatography with ethyl acetate/petroleum ether (1: 8, v/v) as eluent to afford **2** as a white solid (260.0 mg, 55.6%). ^1^H-NMR (500 MHz, DMSO*-d_6_*) δ 7.42 – 7.38 (m, 2H), 7.35 – 7.32 (m, 3H), 7.29 – 7.26 (m, 3H), 7.24 – 7.20 (m, 5H), 7.19 (d, *J* = 2.7 Hz, 1H), 6.82 (dd, *J* = 9.0, 1.5 Hz, 4H), 3.72 (s, 3H), 3.70 (t, *J* = 4.9 Hz, 4H), 3.11 (t, *J* = 4.9 Hz, 4H).^13^C NMR (125 MHz, DMSO) δ 158.35, 150.93, 143.90, 137.54, 131.64, 131.23, 129.59, 129.43, 129.27, 129.03, 128.85, 128.03, 127.60, 121.20, 114.33, 114.06, 66.46, 55.44, 48.00.HRMS: m/z calcd for C_32_H_29_N_3_O ([M + H]^+^) 488.2338, found 488.2380.

**Synthesis of compound 3**

The compound 2 (240.0 mg, 0.49 mmol) was dissolved in a mixture of acetic acid (2.5 mL) and 48% aqueous hydrobromic acid (6.0 mL), then heated to reflux for overnight. The reaction was quenched with ice water followed by adjusting pH value to neutral with sodium hydroxide. Then the mixture was extracted with ethyl acetate, dried over dried over anhydrous magnesium sulfate and concentrated by vacuum-rotary evaporation procedure. The crude product was purified by silica column chromatography with DCM/MeOH (40: 1, v/v) as eluent to obtain **3** as a pink solid (165.0 mg, 71.1%). ^1^H NMR (400 MHz, DMSO*-d_6_*) δ 9.34 (s, 1H), 7.35 – 7.32 (m, 3H), 7.29 (d, *J* = 2.2 Hz, 1H), 7.27 (t, *J* = 2.9 Hz, 4H), 7.24 – 7.18 (m, 5H), 7.18 – 7.16 (m, 1H), 6.84 – 6.80 (m, 2H), 6.65 – 6.61 (m, 2H), 3.72 – 3.67 (m, 4H), 3.14 – 3.08 (m, 4H). HRMS: m/z calcd for C_31_H_27_N_3_O_2_ ([M + H]^+^) 474.2182, found 474.2169.

**Synthesis of compound 4**

Hexamethylenetetramine (53.21 mg, 0.38 mmol) was dissolved in 6 mL trifluoroacetic acid in a dried round-bottomed flask equipped with magnetic stirring bar. To the mixture was added compound 3 (150.0 mg, 0.32 mmol) and stirred at 90 ℃ for 4 h. After cooling to room temperature, quenched reaction with water and the pH value was adjusted to neutral with sodium hydroxide. Then the mixture was extracted with dichloromethane, dried over anhydrous magnesium sulfate, concentrated by rotary evaporation. Further purification was conducted on silica gel chromatography with DCM/MeOH (80: 1, v/v) as eluent to obtain **4** as a canary yellow solid (70.0 mg, 43.8 %). ^1^H NMR (500 MHz, DMSO*-d_6_*) δ 10.66 (s, 1H), 10.21 (s, 1H), 7.88 (d, *J* = 2.2 Hz, 1H), 7.50 (dd, *J* = 8.6, 2.3 Hz, 1H), 7.37 – 7.30 (m, 3H), 7.30 – 7.26 (m, 3H), 7.25 – 7.19 (m, 6H), 6.83 (dd, *J* = 20.2, 8.8 Hz, 3H), 3.73 – 3.64 (m, 4H), 3.16 – 3.02 (m, 4H). ^13^C NMR (125 MHz, DMSO) δ 191.20, 159.40, 150.52, 146.26, 136.96, 135.60, 134.09, 131.06, 130.35, 129.96, 129.16, 128.99, 128.80, 128.64, 128.46, 128.28, 126.59, 126.24, 122.15, 120.55, 117.03, 113.85, 65.98, 47.50. HRMS: m/z calcd for C_32_H_27_N_3_O_3_ ([M + H]^+^) 502.2131, found 502.3580.

**Synthesis of ASMP-AP**

Compound 4 (45.0 mg, 0.09 mmol) was dissolved in 8 mL absolute alcohol and then 2-aminothiophenol (22.5 mg, 0.18 mmol) was added under stirring. To the system was added a few drops of hydrogen peroxide (30%) and hydrochloric acid, and the reaction was performed at room temperature for 2 h. Quenched reaction with water and extracted with ethyl acetate. The combined organic extracts were washed with brine, dried over anhydrous magnesium sulfate and evaporated. The crude product was purified by silica column chromatography using ethyl acetate/petroleum ether (1: 8, v/v) as eluent to afford **ASMP-AP** as a brown solid (28 mg, 51.3 %). ^1^H NMR (500 MHz, DMSO*-d_6_*) δ 11.59 (s, 1H), 8.40 (s, 1H), 8.13 (d, *J* = 7.9 Hz, 1H), 8.05 (d, *J* = 8.1 Hz, 1H), 7.57 – 7.53 (m, 2H), 7.48 – 7.43 (m, 2H), 7.40 (dd, *J* = 8.6, 2.2 Hz, 2H), 7.37 – 7.34 (m, 3H), 7.33 (d, *J* = 2.9 Hz, 2H), 7.28 (d, *J* = 3.6 Hz, 1H), 7.27 (d, *J* = 2.5 Hz, 2H), 7.25 (d, *J* = 3.9 Hz, 1H), 6.94 (d, *J* = 8.6 Hz, 1H), 6.85 (d, *J* = 8.9 Hz, 2H), 3.71 (t, *J* = 4.8 Hz, 4H), 3.15 – 3.11 (m, 4H). ^13^C NMR (125 MHz, DMSO) δ 166.00, 155.46, 153.81, 151.91, 151.03, 146.75, 140.72, 139.56, 137.48, 136.29, 134.60, 131.62, 130.87, 130.46, 129.53, 129.28, 129.01, 128.77, 126.81, 125.65, 124.58, 123.62, 123.18, 122.58, 121.11, 118.35, 117.26, 116.05, 114.38, 66.47, 55.37. HRMS: m/z calcd for C_38_H_30_N_4_O_2_S ([M + H]^+^) 607.2168, found 607.2135.

# Theoretical ESIPT analysis

**
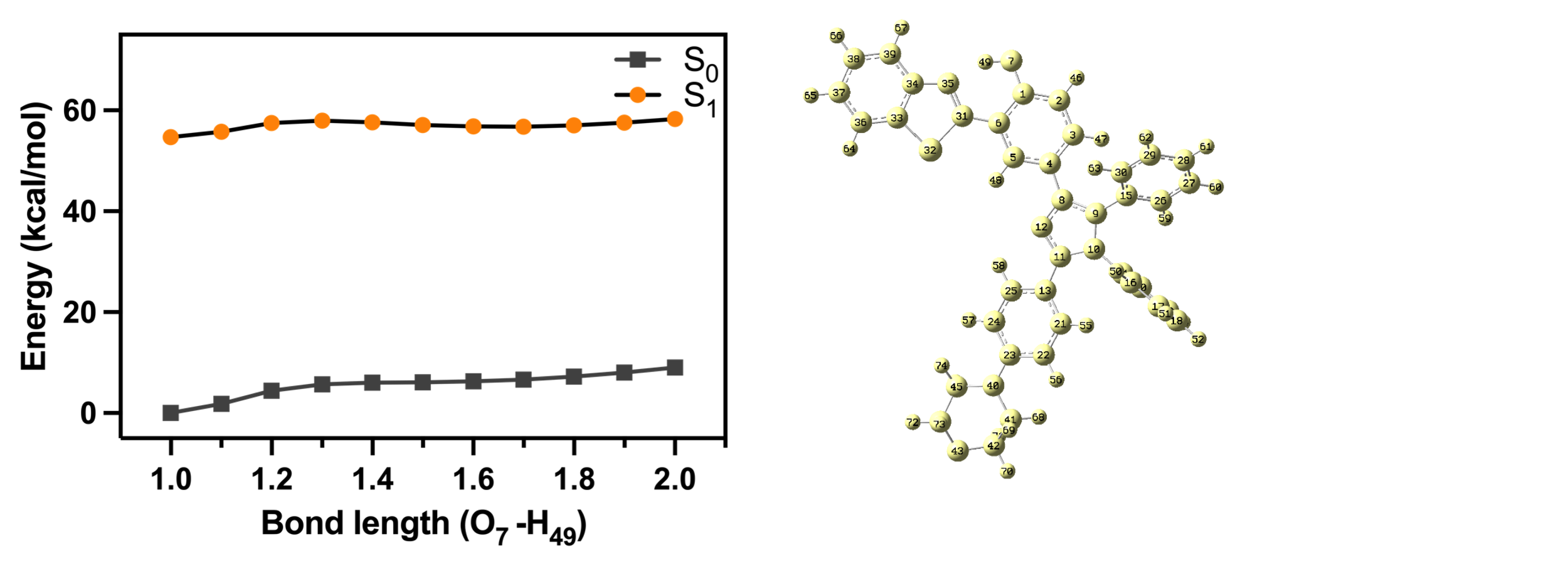
**

**Figure S1.** The constructed potential energy scan surface of both S0 and S1 states of **ASMP-AP** as a function of O_7_-H_49_.

# Optical properties and biological studies

**
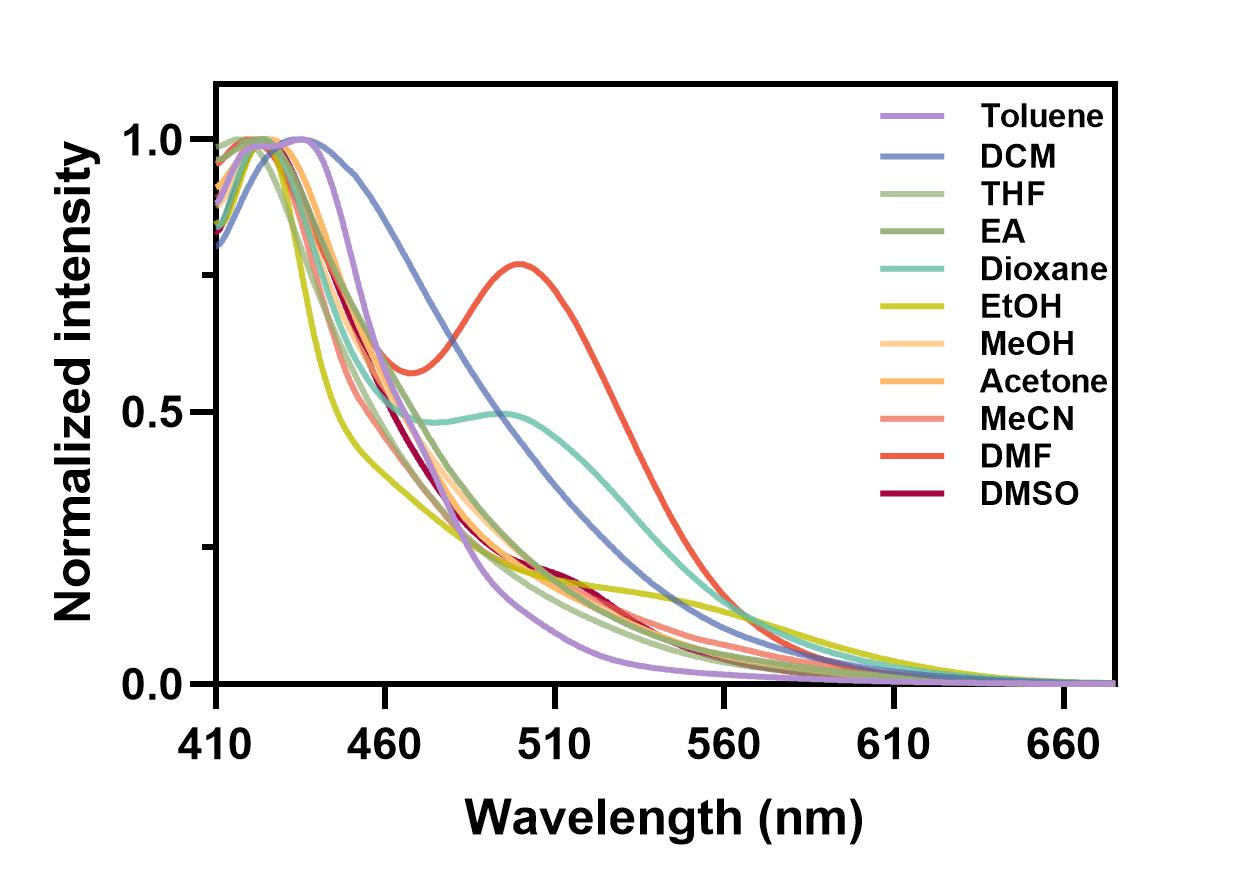
**

**Figure S2.** Fluorescence spectra of **ASMP-AP** (10µM) in different solvents. *λ*_ex_ = 375 nm

**
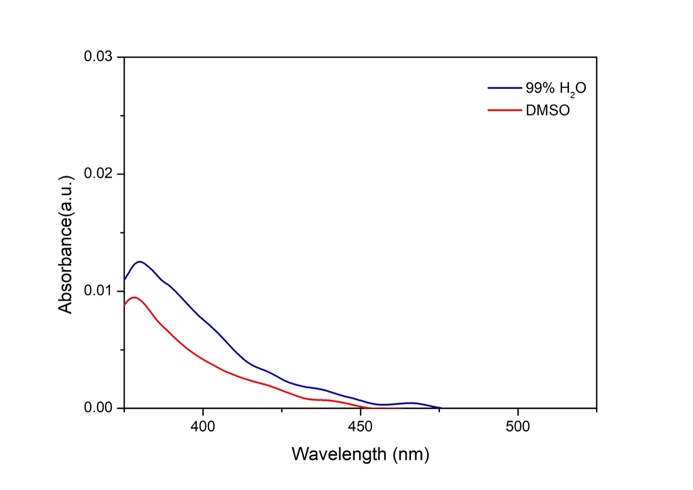
**

**Figure S3.** Absorption spectra of **ASMP-AP** (10µM) in DMSO and in aqueous solution (1% DMSO).

**
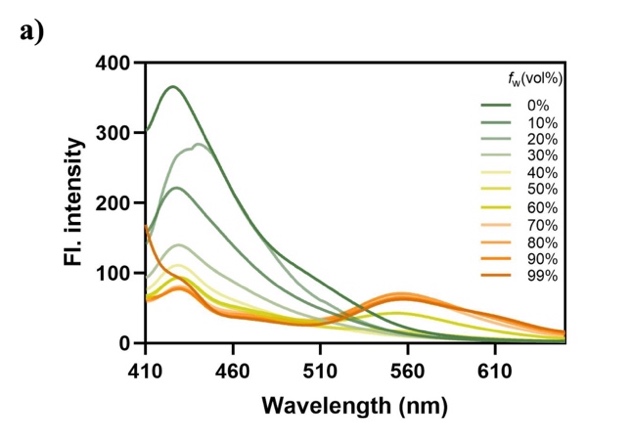

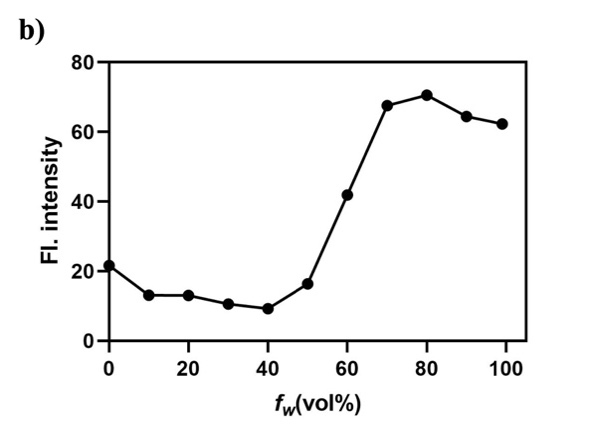
**

**Figure S4.** AIE properties of **ASMP-AP**. a) Fluorescence spectra of **ASMP-AP** (10 µM) in DMSO/water mixtures with different volume fraction of water (*f_w_*). b) Fluorescence emission at 560 nm of **ASMP-AP** (10 µM) in DMSO/water mixed solvent. *λ*_ex_ = 375 nm.


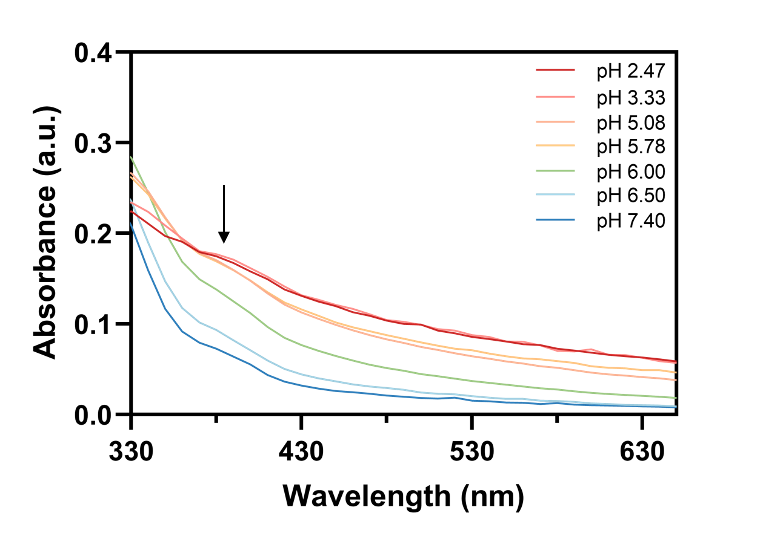


**Figure S5**. Absorption spectra of **ASMP-AP** in aqueous solution (10% DMSO) with different pH.


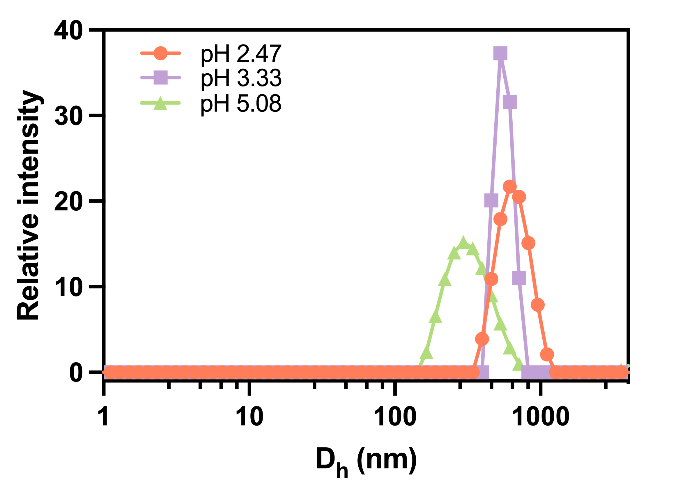


**Figure S6**. DLS spectra of **ASMP-AP** (10 µM) in PBS buffer at different pH values.


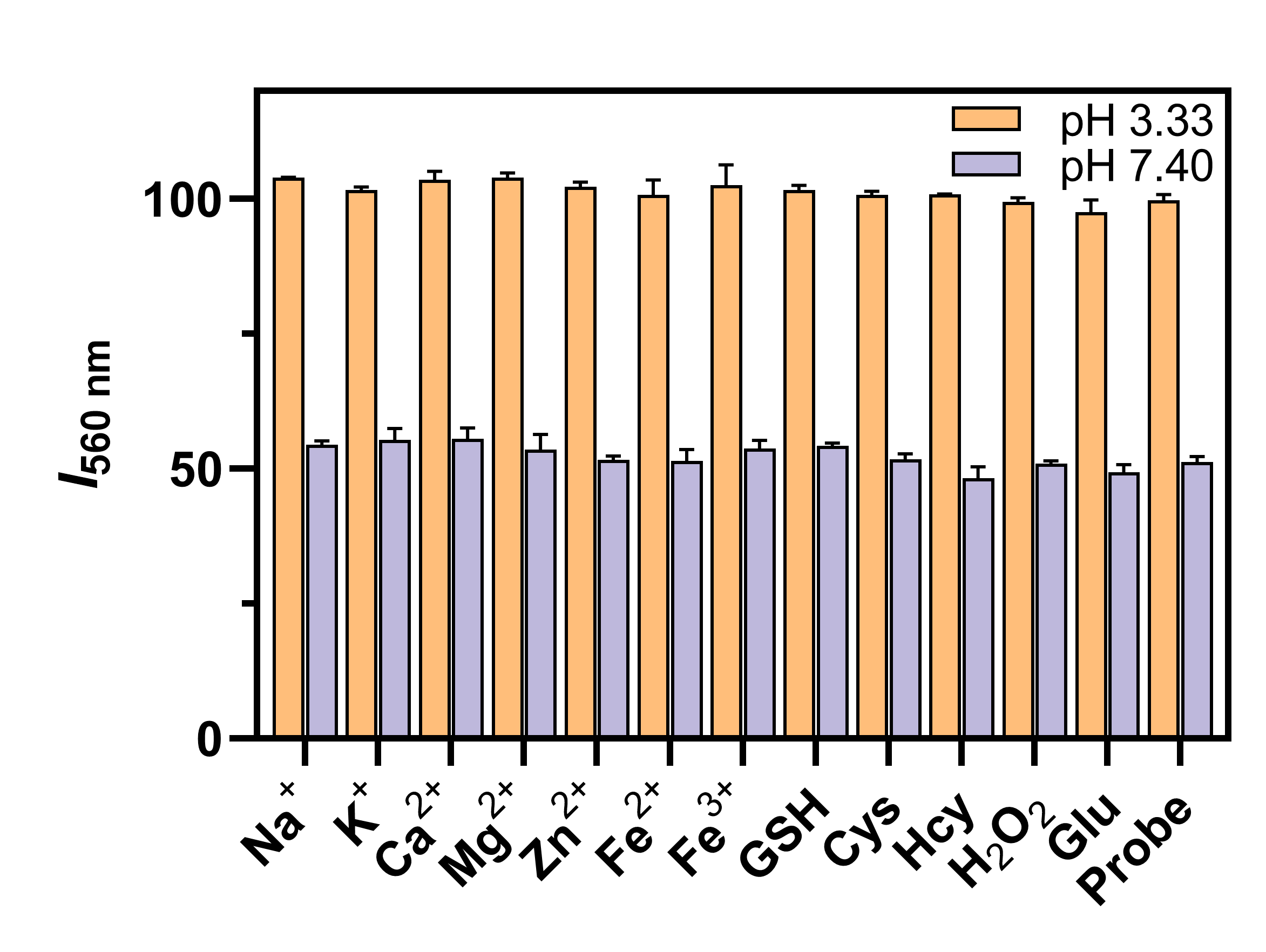


**Figure S7**. Fluorescence responses of **ASMP-AP** (10 µM) to pH 3.33 and 7.40 in the absence and presence of possible interfering substances at 37 °C, respectively. Such as metal ions (100 µM), essential thiols (1 mM), H_2_O_2_ (100 µM).


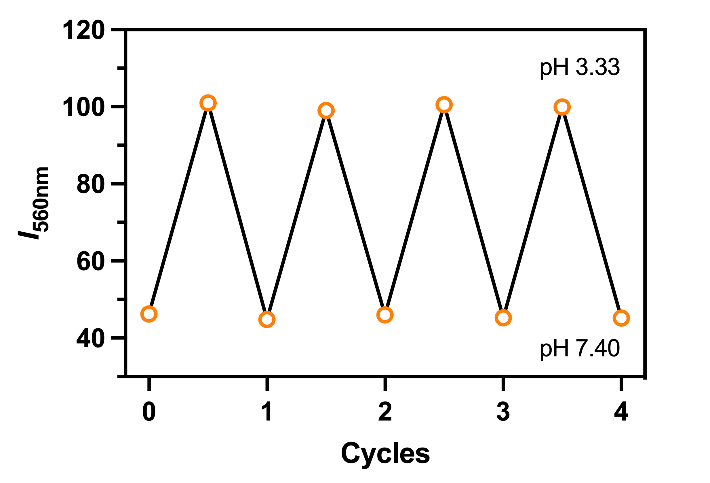


**Figure S8**. Emission maximum versus switching cycles between pH 3.33 and pH 7.4.


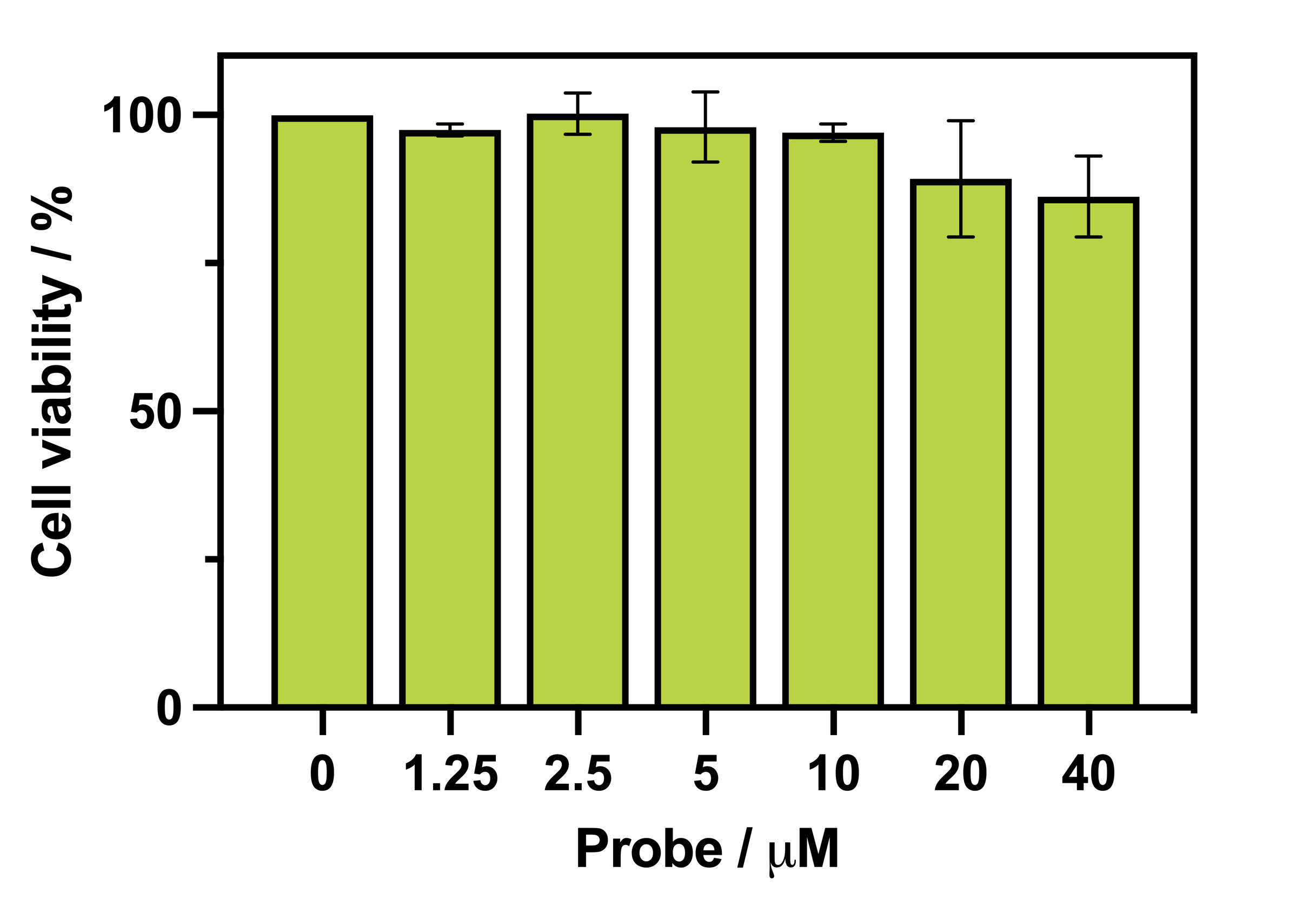


**Figure S9**. Cytotoxicity of ASMP-AP to Hela cells determined by MTT assay. The data are based on the average and show the standard deviation (n = 3).


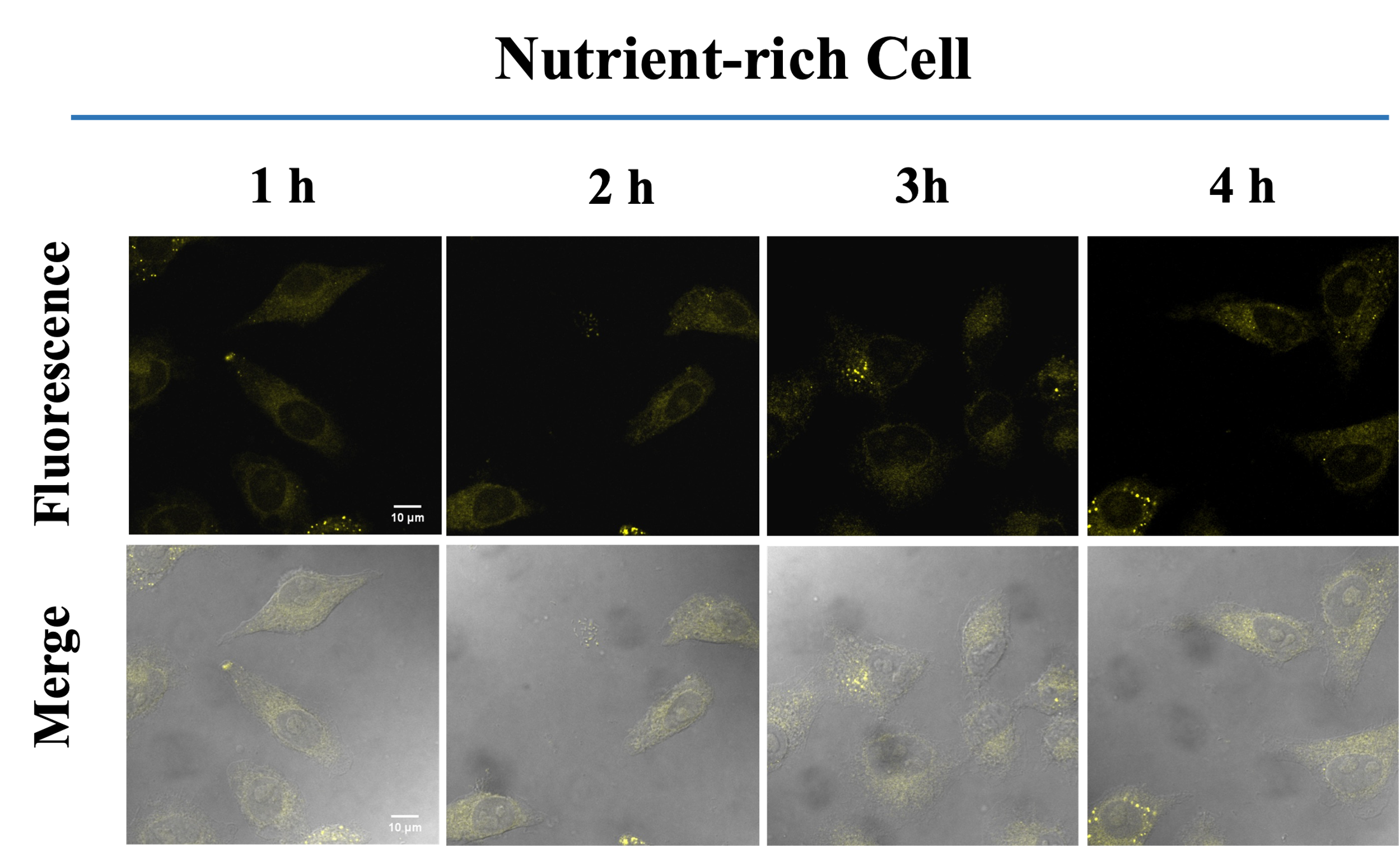


**Figure S10**. CLSM images of cells in in nutrient-rich medium at different time nodes. *λ*_ex_ = 405 nm; *λ*_em_ = 560-620 nm. Scale bars, 10 μm.


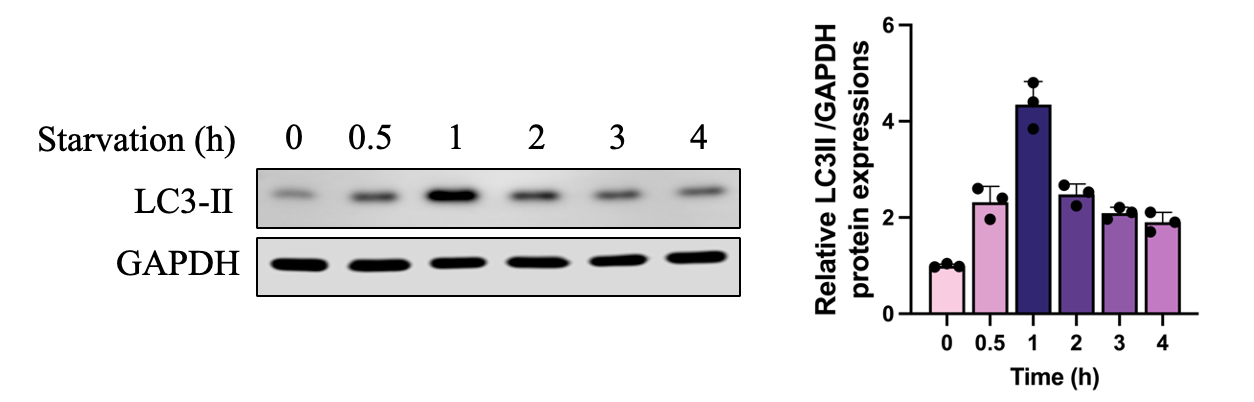


**Figure S11**. Autophagy-related protein expression and statistical analysis under starvation conditions.


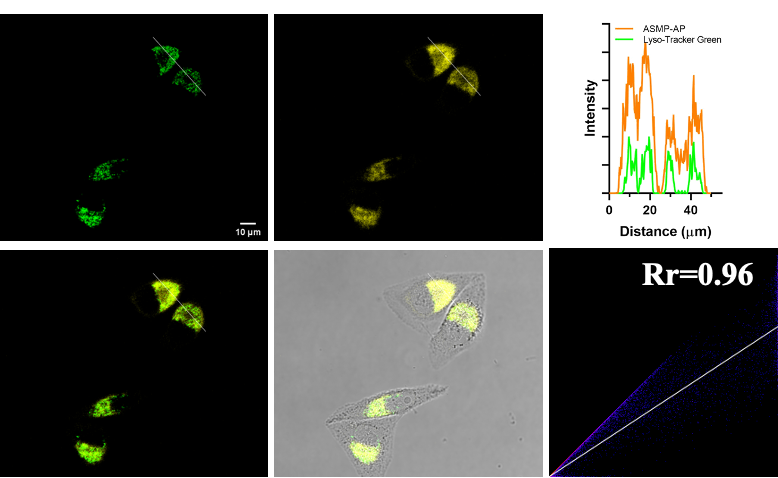


**Figure S12**. Lysosome-targeting properties of **ASMP-AP** in HeLa cells after 4 h-starvation treatment. Colocalization images stained with LysoTracker Green (1.0 μM, green channel, *λ*_ex_ = 488 nm, *λ*_em_ = 500-550 nm) and **ASMP-AP** (10 μM, yellow channel, *λ*_ex_ = 405 nm, *λ*_em_ = 560-620 nm), and the correlation of **ASMP-AP** and LysoTracker Green intensities as well as the intensity profiles within the ROI. Scale bars, 10 µm.


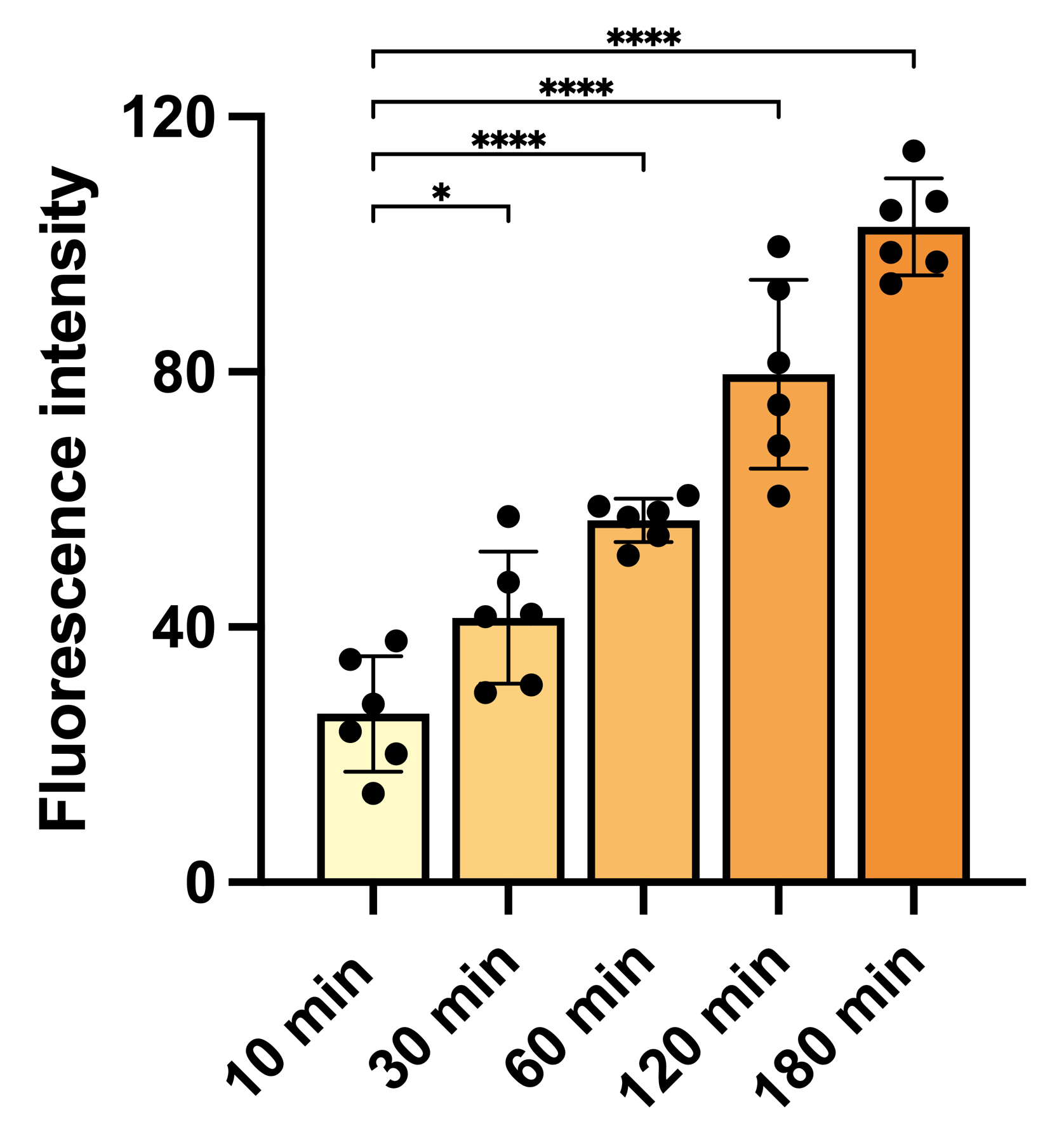


**Figure S13**. Analysis of the fluorescence intensity of **ASMP-AP** channel in panel Figure 6A (n = 6, by ImageJ software). Statistical analyses were performed with One-way Anova. *: *p* < 0.05, ****: *p* < 0.0001.


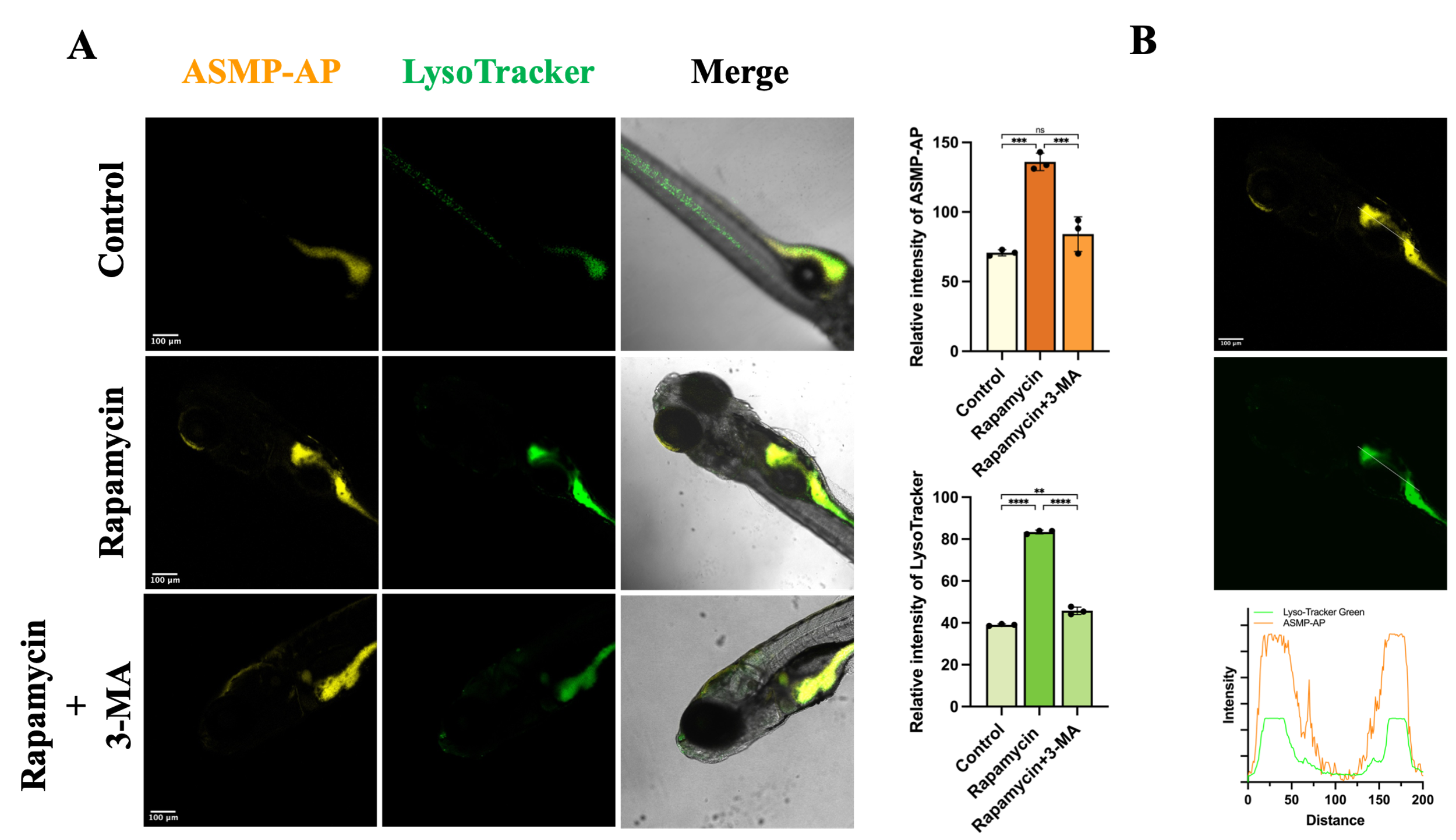


**Figure S14**. Confocal fluorescence images of zebrafish larvae in rapamycin induced autophagy. A) Followed by the pretreatment with rapamycin for 12 h at 28 ℃, zebrafish larvae were stained with **ASMP-AP** (10 μM, *λ*_ex_ = 405 nm, *λ*_em_ = 560-620 nm) and LysoTracker green (1.0 μM, *λ*_ex_ = 488 nm, *λ*_em_ = 500-550 nm). Statistical analyses were performed with One-way Anova. *: *p* < 0.05, **: *p* < 0.01, ***: *p* < 0.001, and ****: *p* < 0.0001. B) Colocalization relationship of **ASMP-AP** with LysoTracker green. Scale bars, 100 µm.


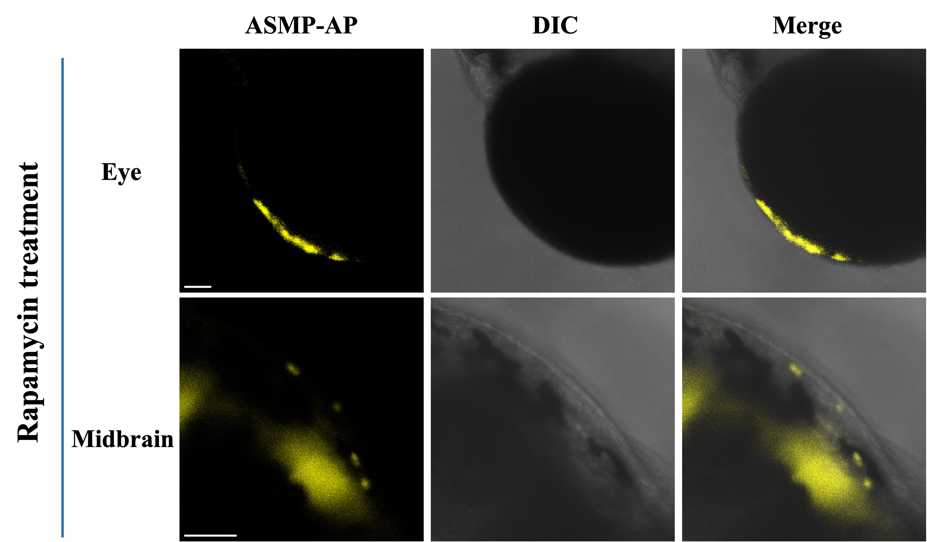


**Figure S15**. Confocal fluorescence images of different positions of zebrafish larvae (eyes and midbrain) with rapamycin treatment. **ASMP-AP** (10 μM, *λ*_ex_ = 405 nm, *λ*_em_ = 560-620 nm). Scale bars, 100 µm.

**Table S1** Comparison of properties of various pH-sensitive AIE probes.

| Probes | λ_em_ (nm) | pH sensing ranges | Stokes shift (nm) | Mechanism | Ref. |
| --- | --- | --- | --- | --- | --- |
| **** | 489/615 | 4.7-8.0 | 185 | The protonation/deprotonation of the sulfonate group endow the pH-induced hydrophobicity change and particle formation. | [[2](#_ENREF_2)] |
|  | 484/551 | 6.9-8.0 | 184 | The protonation of carboxyl and phenolic hydroxyl groups results in low solubility and an aggregation. | [[3](#_ENREF_3)] |
|  | 503/615 | 2.6-6.8 | 144 | The enhanced ICT induced by the protonation of the pyridinyl group forms the basis of the ratiometric fluorescence pH sensor. | [[4](#_ENREF_4)] |
|  | 790 | 5.0-10.7 | 484 (Pseudo-Stokes shift) | The cassette becomes less aggregated in basic aqueous solution because of charge repulsion among three negative phenolate ions. | [[5](#_ENREF_5)] |
|  | 743 | 4.0-7.0 | 83 | The phenolic form with lower solubility is generated with pH increase so that activates the AIE response. | [[6](#_ENREF_6)] |
|  | 560 | 1.5-7.4 | 195 | pH-induced different aggregation states rather than the enol/ketone equilibrium cause the fluorescence changes with AIE behavior. | This work |

# Characterization of compounds


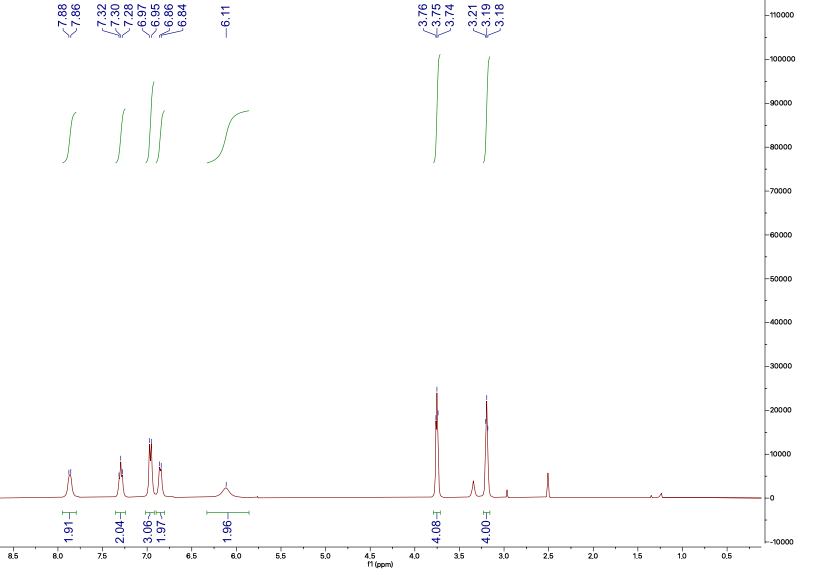


**Figure S16.** ^1^H NMR spectra (DMSO-*d_6_*)) of compound **1**.


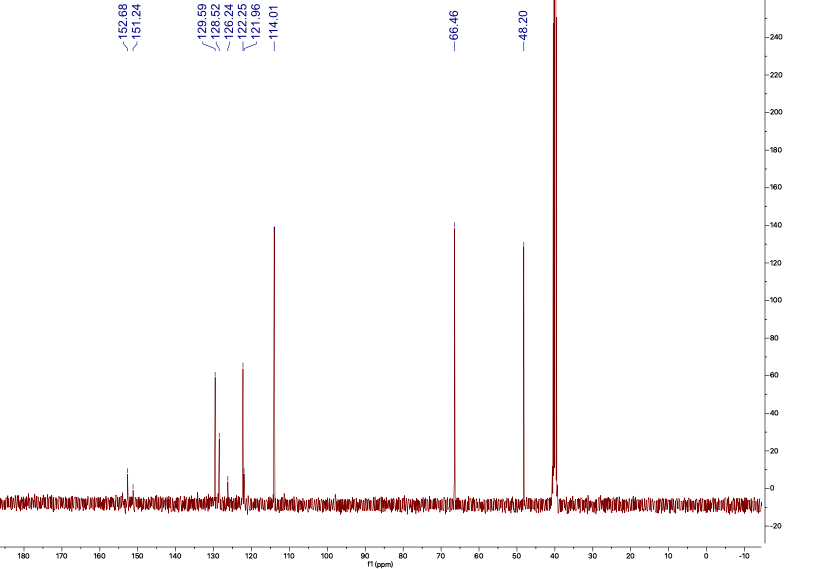


**Figure S17.** ^13^C NMR spectra (DMSO-*d_6_*)) of compound **1**.

**Figure S18.** HRMS spectrum of compound **1**.


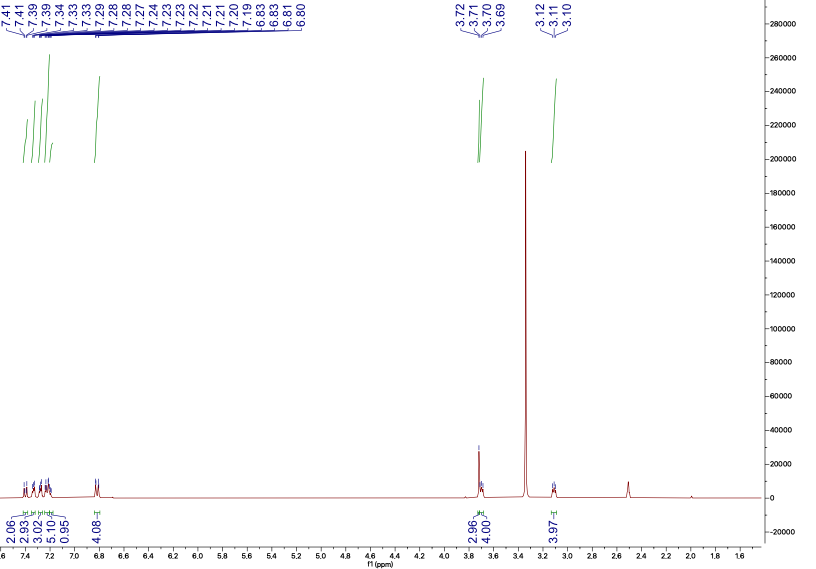


**Figure S19.** ^1^H-NMR spectra (DMSO-*d_6_*) of compound **2**.


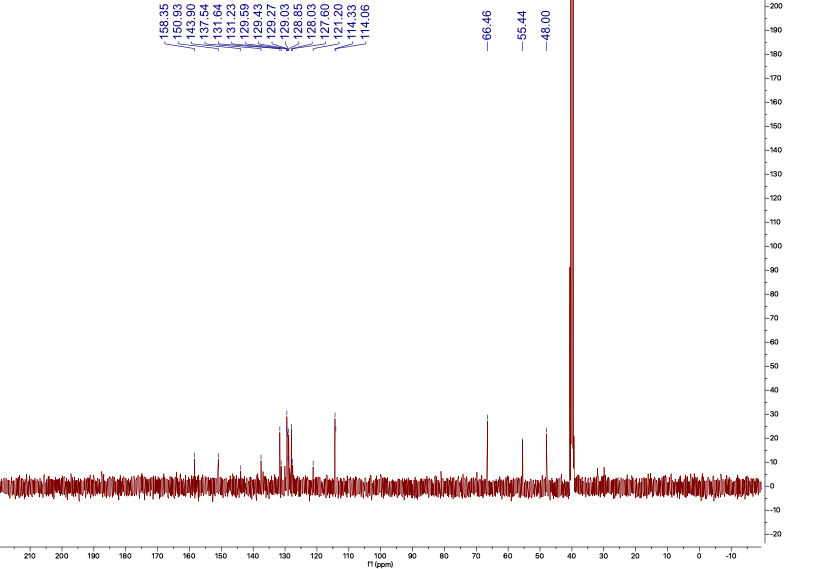


**Figure S20.** ^13^C NMR spectra (DMSO-d6) of compound **2**.

**Figure S21.** HRMS spectrum of compound **2**.


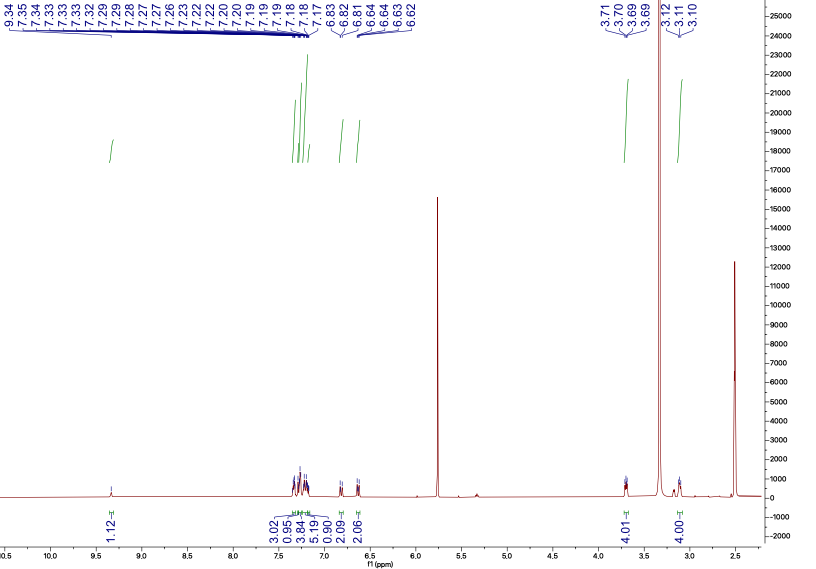


**Figure S22.** ^1^H-NMR spectra (DMSO-*d_6_*) of compound **3**.

**Figure S23.** HRMS spectrum of compound **3**.


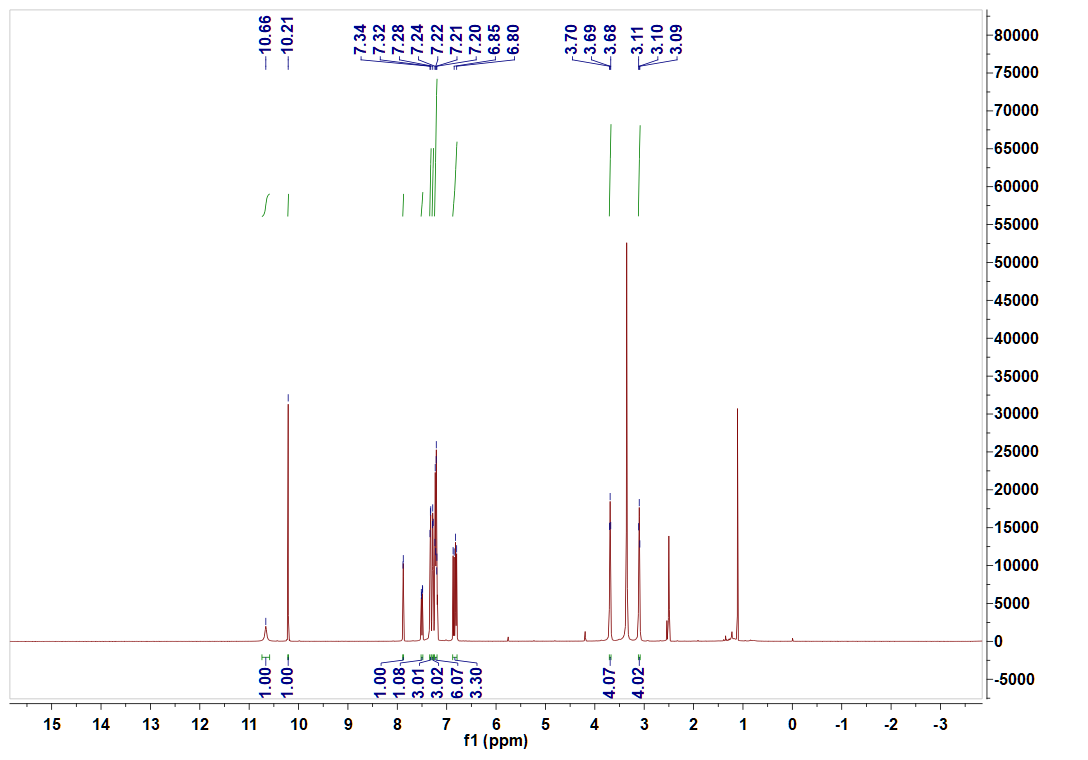


**Figure S24.** ^1^H NMR spectra (DMSO-*d_6_*) of compound **4**.


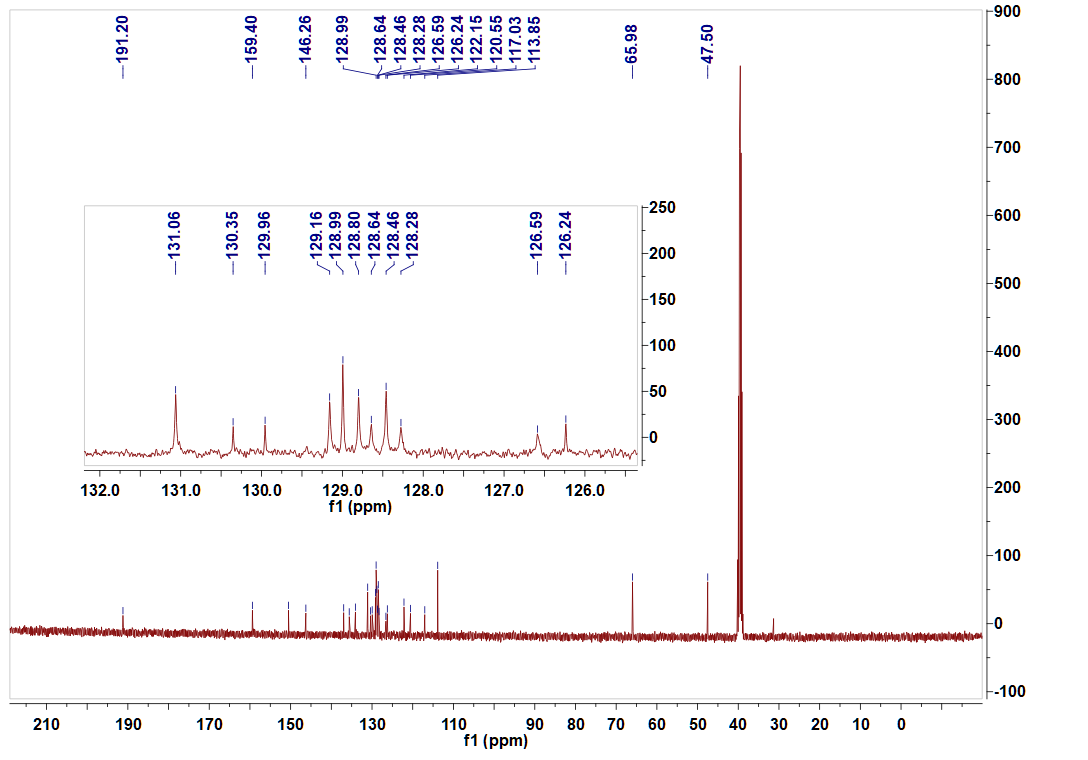


**Figure S25.** ^13^C NMR spectra (DMSO-*d_6_*) of compound **4**.

**Figure S26.** HRMS spectrum of compound **4**.


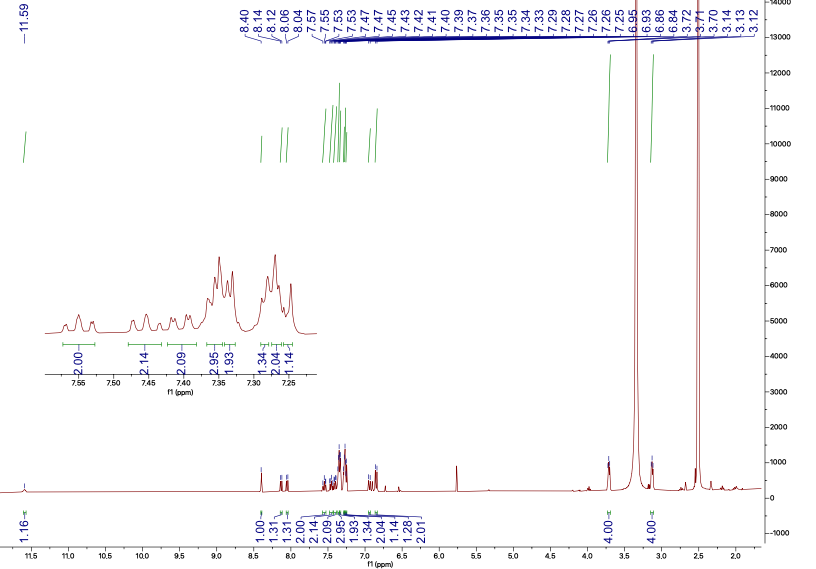


**Figure S27.** ^1^H NMR spectra (DMSO-*d_6_*) of **ASMP-AP**.


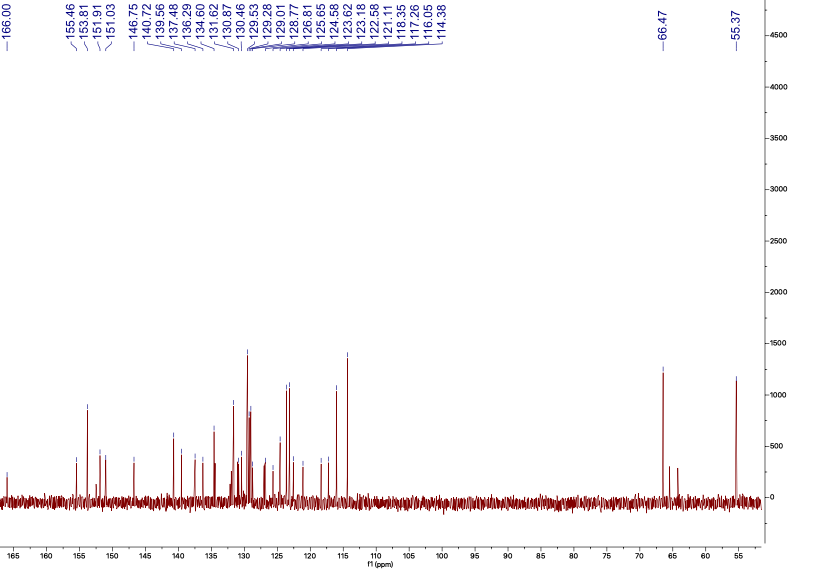


**Figure S28.** ^13^C NMR spectra (DMSO-*d_6_*) of **ASMP-AP**.

**Figure S29.** HRMS spectrum of compound **ASMP-AP**.

**References**

1. Humphrey W, Dalke A, Schulten K. VMD: visual molecular dynamics. Journal of molecular graphics*.* 1996; 14:33-38, 27-38.

2. Chen S, Hong Y, Liu Y, Liu J, Leung CWT, Li M, et al. Full-Range Intracellular pH Sensing by an Aggregation-Induced Emission-Active Two-Channel Ratiometric Fluorogen. J Am Chem Soc*.* 2013; 135:4926-4929.

3. Li K, Feng Q, Niu G, Zhang W, Li Y, Kang M, et al. Benzothiazole-Based AIEgen with Tunable Excited-State Intramolecular Proton Transfer and Restricted Intramolecular Rotation Processes for Highly Sensitive Physiological pH Sensing. ACS Sens*.* 2018; 3:920-928.

4. Shi X, Yan N, Niu G, Sung SHP, Liu Z, Liu J, et al. In vivo monitoring of tissue regeneration using a ratiometric lysosomal AIE probe. Chem Sci*.* 2020; 11:3152-3163.

5. Fang M, Xia S, Bi J, Wigstrom TP, Valenzano L, Wang J, et al. A cyanine-based fluorescent cassette with aggregation-induced emission for sensitive detection of pH changes in live cells. Chem Commun*.* 2018; 54:1133-1136.

6. Zhao X, Chen Y, Niu G, Gu D, Wang J, Cao Y, et al. Photostable pH-Sensitive Near-Infrared Aggregation-Induced Emission Luminogen for Long-Term Mitochondrial Tracking. ACS Appl Mater Interfaces*.* 2019; 11:13134-13139.
